# Genome-wide analysis suggests a differential microRNA signature associated with normal and diabetic human corneal limbus

**DOI:** 10.1038/s41598-017-03449-7

**Published:** 2017-06-14

**Authors:** Mangesh Kulkarni, Aleksandra Leszczynska, Gabbie Wei, Michael A. Winkler, Jie Tang, Vincent A. Funari, Nan Deng, Zhenqiu Liu, Vasu Punj, Sophie X. Deng, Alexander V. Ljubimov, Mehrnoosh Saghizadeh

**Affiliations:** 1 Biomedical Sciences, Cedars-Sinai Medical Center, Los Angeles, California USA; 2 Regenerative Medicine Institute Eye Program, Cedars-Sinai Medical Center, Los Angeles, California USA; 3 Genomics Core, Cedars-Sinai Medical Center, Los Angeles, California USA; 4 Samuel Oschin Comprehensive Cancer Institute, Cedars-Sinai Medical Center, Los Angeles, California USA; 5 Keck School of Medicine, University of Southern California, Los Angeles, California USA; 6 David Geffen School of Medicine, University of California Los Angeles, Los Angeles, California USA

## Abstract

Small non-coding RNAs, in particular microRNAs (miRNAs), regulate fine-tuning of gene expression and can impact a wide range of biological processes. However, their roles in normal and diseased limbal epithelial stem cells (LESC) remain unknown. Using deep sequencing analysis, we investigated miRNA expression profiles in central and limbal regions of normal and diabetic human corneas. We identified differentially expressed miRNAs in limbus vs. central cornea in normal and diabetic (DM) corneas including both type 1 (T1DM/IDDM) and type 2 (T2DM/NIDDM) diabetes. Some miRNAs such as miR-10b that was upregulated in limbus vs. central cornea and in diabetic vs. normal limbus also showed significant increase in T1DM vs. T2DM limbus. Overexpression of miR-10b increased Ki-67 staining in human organ-cultured corneas and proliferation rate in cultured corneal epithelial cells. MiR-10b transfected human organ-cultured corneas showed downregulation of PAX6 and DKK1 and upregulation of keratin 17 protein expression levels. In summary, we report for the first time differential miRNA signatures of T1DM and T2DM corneal limbus harboring LESC and show that miR-10b could be involved in the LESC maintenance and/or their early differentiation. Furthermore, miR-10b upregulation may be an important mechanism of corneal diabetic alterations especially in the T1DM patients.

## Introduction

Many forms of corneal conditions can be sight threatening, such as diabetic keratopathy and limbal stem cell deficiency (LSCD) that is hard to treat even with corneal transplantation. Such diseases are related to abnormal self-renewal and wound healing due to epithelial dysfunction. Corneal epithelium is constantly renewed by limbal epithelial stem cells (LESC) that in humans exclusively reside in the corneoscleral junction, limbus^1–3^. LESC have lifetime capacity for self-renewal, and the ability to generate transient amplifying (TA) cells, which in turn differentiate into central corneal epithelium. Epithelial cells must remain in homeostasis to ensure normal epithelial function. This includes maintenance, self-renewal and differentiation, which are important parts of the life cycle of corneal epithelium.

In recent years, there has been growing understanding of the dynamic role played by microRNAs (miRNAs) in modulating tissue homeostasis. MiRNAs are evolutionarily conserved small noncoding RNAs that regulate gene expression post-transcriptionally by binding to complementary sequences on 3′UTR of target mRNA that usually results in gene silencing by inducing mRNA degradation or inhibition of mRNA translation^4–7^. MiRNAs have a unique multi-targeted mode of action enabling each miRNA to regulate the expression of multiple genes in contrast to traditional inhibitors that target one specific protein^6, 7^. Many recent studies have shown the significant role of miRNA in development and maintenance of stem cells, and function during differentiation^8, 9^. Whereas others and we have documented the role of miRNAs in wound healing, tissue maintenance and differentiation^10–17^, few studies have explored the role of miRNAs in LESC homeostasis and corneal diseases^14, 17–21^. Stem or progenitor cells embrace great potential in regenerative medicine for treatment of corneal diseases. Therefore, the mechanistic study of LESC miRNAs could provide us with new insights into the regulatory mechanisms of stem cells in normal and pathological states.

Previously, we reported miRNA expression profiling in the central part of normal and diabetic corneas using microarray analysis^12^. In the present study deep sequencing, which is a more sensitive and quantitative method with broader dynamic range^22^, has been employed to determine the miRNA expression profiles in human limbal compartment and central cornea in normal, as well as in insulin dependent diabetes mellitus (IDDM/T1DM) and non-insulin dependent diabetes mellitus (NIDDM/T2DM). We aimed at identifying differentially expressed miRNAs in human limbal epithelial cells (LEC) vs. mature central epithelium in normal and diabetic corneas, as well as detecting miRNAs dysregulated in diabetes. We also examined how certain limbal-enriched and diabetes-increased miRNAs function in regulatory pathways involved in corneal epithelial self-renewal and differentiation in normal and disease states.

To our knowledge, this is the first study using genome-wide microRNA expression profiling in normal vs. T1DM and T2DM limbal and central human corneas by high throughput sequencing. We also provide novel data on the role of miR-10b, one of the most abundant limbal miRNAs, in the corneal epithelium with possible function in LEC homeostasis.

## Results

### Deep sequencing revealed miRNA expression profiles of normal and diabetic corneas

#### Library integrity and mapping

Library integrity was determined by mapping all reads between 20–30 nt to human genome. MiRNAs were identified and quantified by simultaneously mapping reads to human genome and miRBase. Unmapped sequences were neither repetitive nor represented unknown transcripts, nor were present due to contamination as they rarely mapped against RepBase^23^, NCBI UniGen cluster or non-human genomes. On an average >75% of ncRNA was found to be microRNAs.

#### Differentially expressed miRNAs in limbus vs. central in normal and diabetic corneas

**Figure 1:**
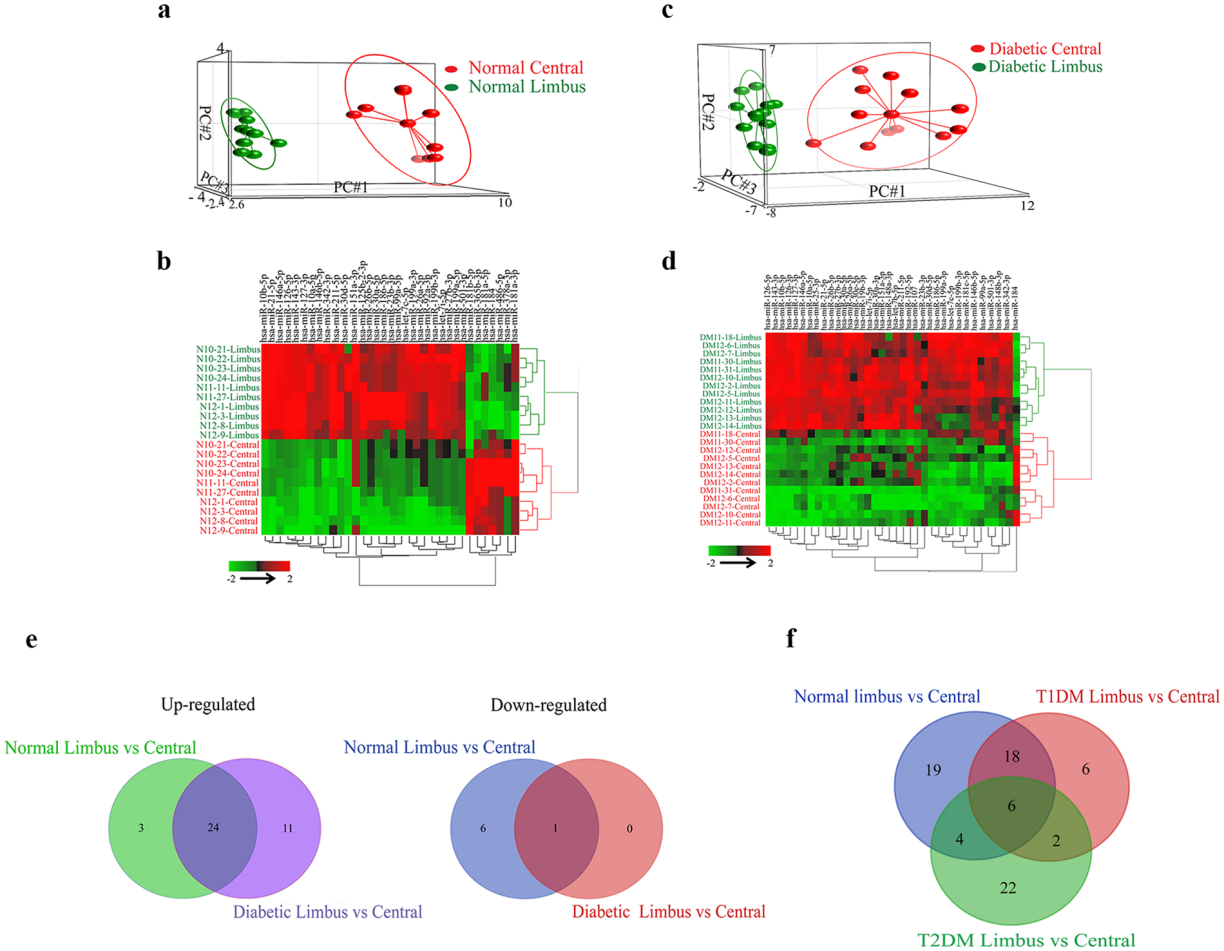
Differentially expressed limbus vs. central miRNAs in normal and diabetic corneas. (**a**–**d**) PCA and two-way hierarchical clustering plots of limbus vs. central cornea. (**a**,**b**) 34 differentially expressed miRNAs in normal limbus vs. central cornea. (**c**,**d**) 36 differentially expressed miRNAs in diabetic limbus vs. central cornea. t-test, FDR adjusted p < 0.05. (**e**) Venn diagrams of up-regulated (left) and down-regulated miRNAs (right) in two-group comparisons of differentially expressed miRNAs (adjusted p < 0.05) in normal and diabetic limbus vs. central corneas. (**f**) Venn diagram of differentially expressed limbus vs. central miRNAs in three-group comparisons in normal, T1DM (IDDM) and T2DM (NIDDM).

After filtering miRNAs as detailed in Materials and Methods, a total of 192 miRNAs were analyzed for further differential expression in a two-group analysis. A set of 47 miRNAs (Supplementary Table S1) was identified as differentially expressed in normal limbus vs. central cornea with a raw p < 0.05 and fold change of ±1.4. Of these 47 miRNAs 34 miRNAs showed a false discovery rate (FDR)-adjusted p ≤ 0.05. A principal component analysis (PCA) and hierarchical clustering showed a clear distinction between normal limbus vs. central cornea (Fig. 1a and b). Similarly, a set of 36 miRNAs with FDR-adjusted p ≤ 0.05 showed a distinct grouping into diabetic limbus vs. central cornea (Fig. 1c and d, and Supplementary Table S2). There were 35 miRNAs upregulated and just one miRNA downregulated in diabetic limbus vs. central cornea (Fig. 1d and Supplementary Table S2). Of 34 differentially expressed miRNAs in normal limbus vs. central cornea (FDR p ≤ 0.05), 27 and 7 miRNAs were upregulated or downregulated, respectively (Fig. 1b and Supplementary Table S1). Venn diagram comparison of differentially expressed limbal vs. central miRNAs in normal and DM corneas revealed 24 common upregulated and one downregulated miRNAs, whereas 11 and three upregulated miRNAs were unique to DM and normal samples, respectively, and six downregulated expressed miRNAs unique to normal samples (Fig. 1e).

PCA and two-way hierarchical clustering of diabetic vs. normal limbal samples revealed 20 differentially expressed miRNAs (Supplementary Table S3), 13 upregulated and seven downregulated in DM vs. normal limbus, (p < 0.05; ±1.3 fold change). Both diabetic groups, T1DM and T2DM, additionally showed distinct differential expression patterns of limbus vs. central cornea. Comparison of normal and T1DM or T2DM diabetic groups revealed that normal samples shared less differentially expressed limbus vs. central miRNAs with T2DM than with T1DM samples, 4 vs. 18 miRNAs (Fig. 1f), suggesting that miRNA expression profiles of T1DM and T2DM groups may be differentially clustered and separated from each other.

#### Differentially expressed miRNAs in T1DM vs. T2DM corneal limbus

**Figure 2:**
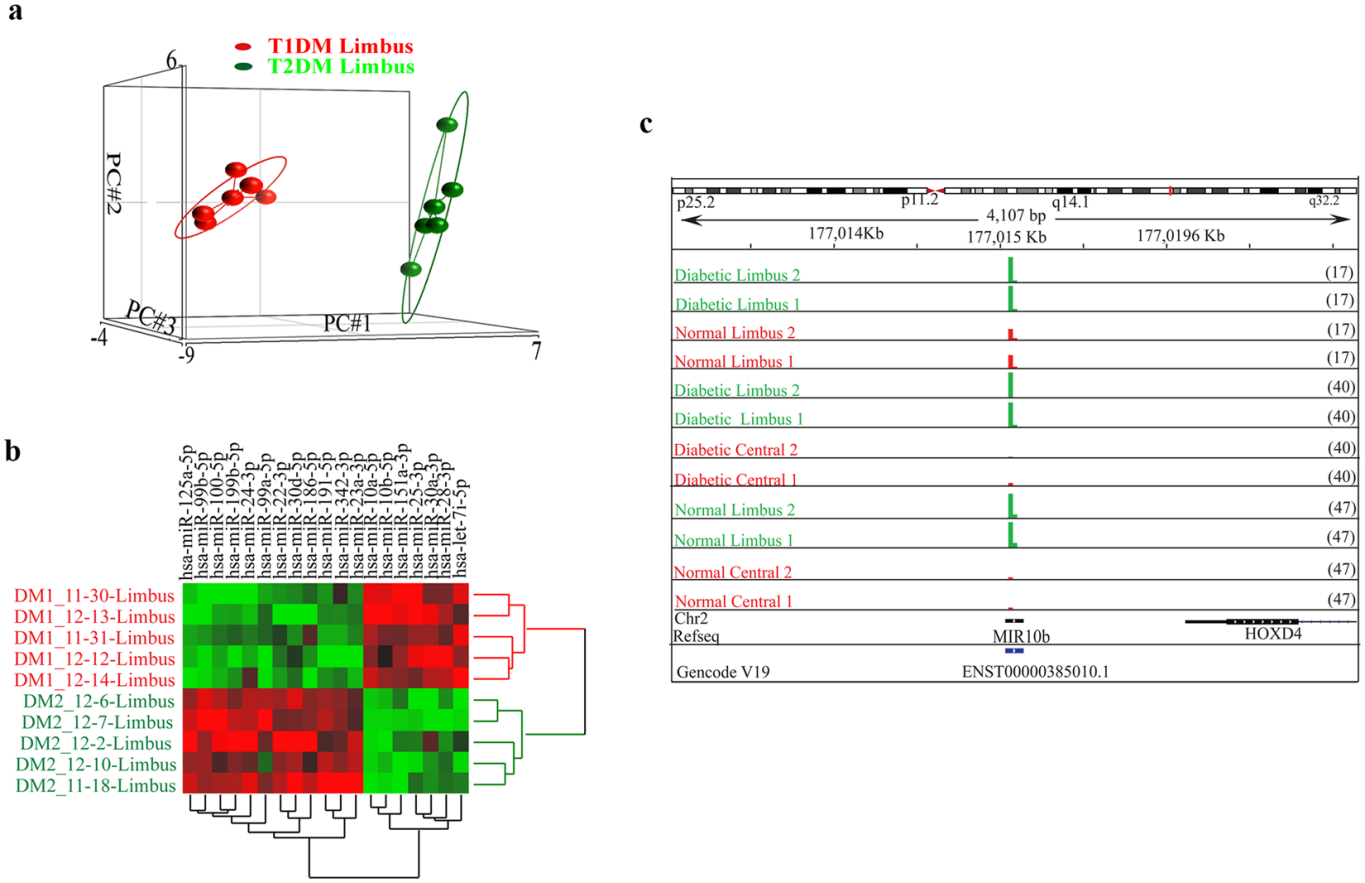
Differentially expressed limbal miRNAs in T1DM vs. T2DM. (**a**) PCA, and (**b**) hierarchical clustering of 19 differentially expressed gene in T1DM (IDDM) vs. T2DM (NIDDM) in limbus, FDR adjusted p < 0.05; (**c**) Genomic snapshot of miR-10b in representative two samples from each two-group comparisons.

Interestingly, PCA and hierarchical clustering showed that the miRNA expression profiles of T1DM and T2DM limbal groups were separated from each other (Fig. 2a and b). Seven miRNAs were upregulated whereas 12 miRNAs were downregulated (1.4-fold cutoff; adjusted p < 0.05) in T1DM vs. T2DM limbus (Supplementary Table S4). Some miRNAs such as miR-10b that was upregulated in limbus vs. central cornea and upregulated in diabetic limbus also showed significant increase in T1DM vs. T2DM limbus. A representative genomic view of the aligned reads for miR-10b in IGV (Integrated genomic viewer) suggests robust increase in the expression of miR10b in normal limbus vs. central as well as in diabetic vs. normal limbus (Fig. 2c) supporting the bioinformatics approach detailed here.

### Validation of differentially expressed miRNAs using QRT-PCR and *in situ* hybridization

**Figure 3:**
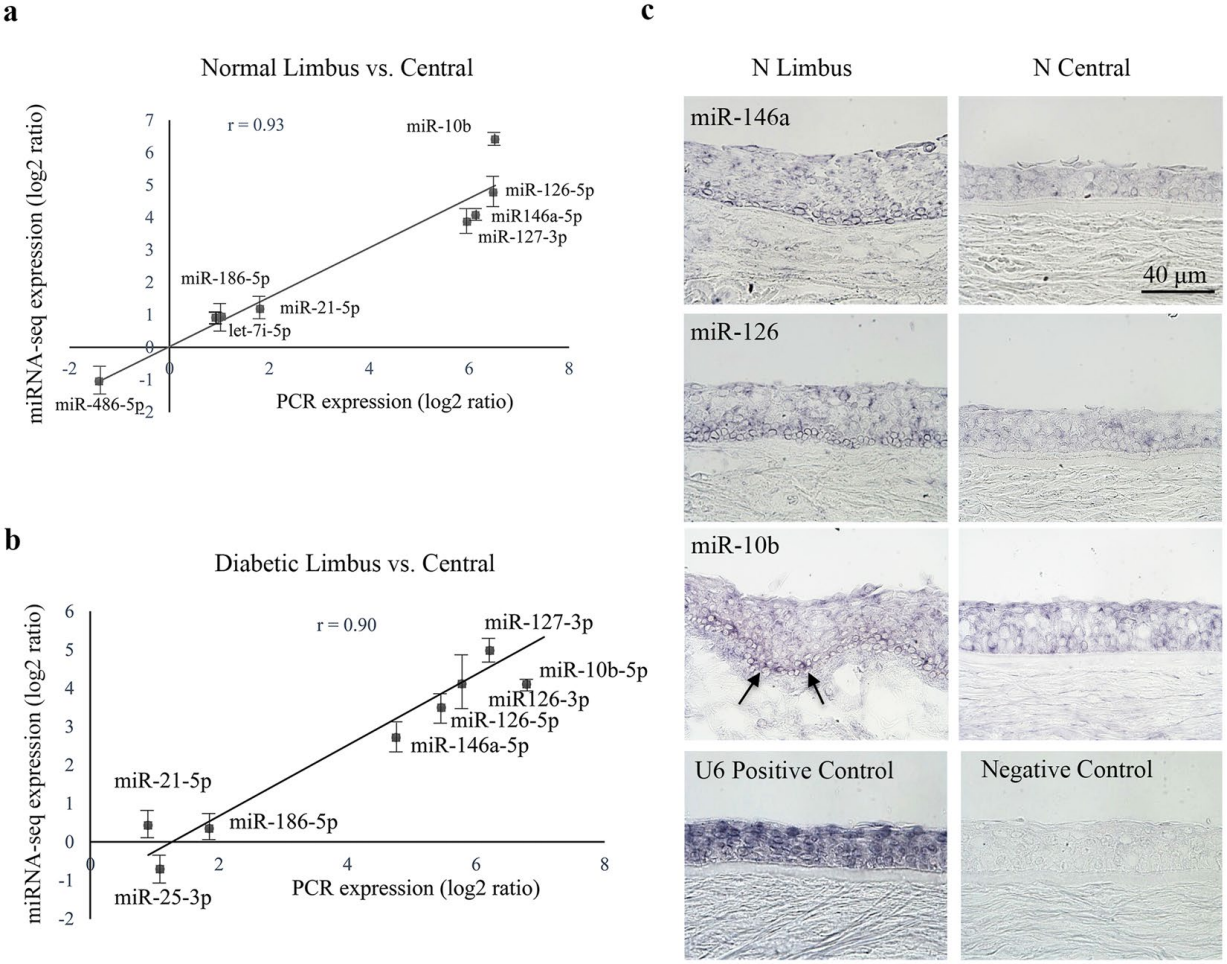
Validation of deep sequencing data of differentially expressed limbus vs. central corneal miRNAs. (**a**,**b**) Quantitative RT-PCR validation of deep sequencing data, limbus vs. central cornea. Several miRNAs are differentially expressed in limbus vs. central cornea. There was a strong concordance between QRT-PCR and deep sequencing data on differentially expressed limbal vs. central miRNAs in normal ((**a**) correlation r = 0.93) and diabetic ((**b**) correlation r = 0.90) samples, Each sample was run in triplicate. (**c**) *In situ* hybridization with locked nucleic acid (LNA) modified probes, miR-146a, miR-126 and miR-10b, was performed on sections of normal corneas, compared to scrambled sequences. The miRNA signals are stronger in the limbus than in the central cornea and more expressed in the basal cell layer in limbal epithelium (﻿a﻿rrows)﻿. U6 miRNA probe and scrambled sequences were used as positive and negative controls, respectively. Bar = 60 μm.

**Figure 4:**
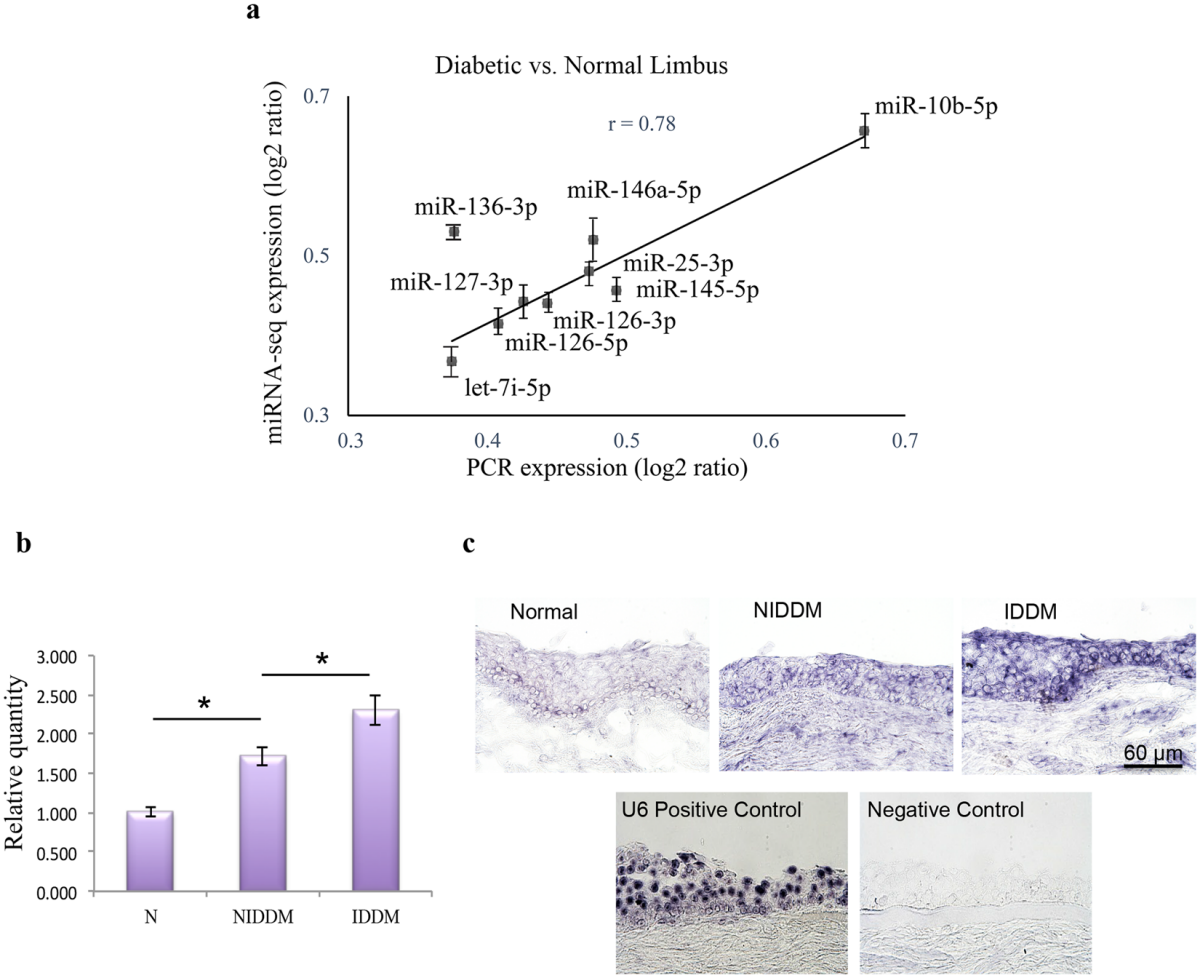
Validation of deep sequencing data of differentially expressed miRNAs in diabetic vs. normal limbus. (**a**) QRT-PCR validation of deep sequencing data, diabetic vs. normal limbus. Several miRNAs are indeed differentially expressed in diabetic vs. normal limbus. The results are in good agreement with deep sequencing analysis (correlation r = 0.78). (**b**) QRT-PCR validation of deep sequencing shows that miR-10b is expressed significantly more in T1DM (IDDM) than in T2DM limbus and more in T2DM (NIDDM) than in normal limbus. (**c**) miR-10b, *in situ* hybridization with LNA modified probes was performed on sections of normal and both T1DM (IDDM) and T2DM (NIDDM) corneas, compared to scrambled sequences. The miRNA signals are stronger in the T1DM than in T2DM limbus and the signals are stronger in T2DM than normal limbus, corroborating our QRT-PCR and deep sequencing data. U6 miRNA probe and scrambled sequences were used as positive and negative controls, respectively. Bar = 60 μm.

The performance of our deep sequencing and bioinformatics approach was validated using quantitative RT-PCR (QRT-PCR). Selected miRNAs differentially expressed in both normal and diabetic limbus vs. central cornea and in diabetic vs. normal limbus were validated by QRT-PCR in independent samples (Supplementary Fig. S1 and Figs 3 and 4). There was a strong concordance between QRT-PCR and deep sequencing data analysis of differentially expressed miRNAs in limbus vs. central in both normal (correlation r = 0.93; Fig. 3a) and diabetic (correlation r = 0.90; Fig. 3b) samples. miR-10b, miR-126, miR-127-3p, miR-186, miR-146a, and miR-21 were elevated in both normal and diabetic limbus vs. central (Supplementary Figs S1a,b and 3a,b), whereas miR-486 was down regulated just in the normal and miR-25-3p was down regulated just in the diabetic limbus vs. central cornea in accordance with deep sequencing data (Fig. 3a and b). *In situ* hybridization with locked nucleic acid (LNA) modified probes, which was performed on sections of normal corneas to localize miRNA expression, further corroborated our sequencing data (Fig. 3c). It showed that the signals for miR-146a, miR-126 and miR-10b were stronger in the limbus than in the central cornea (Fig. 3c).

Further, there was a concordance between QRT-PCR and deep sequencing data in differentially expressed miRNAs in diabetic vs. normal limbal samples (correlation r = 0.78) (Supplementary Fig. S1c and Fig. 4a). Differential expression of miR-10b in T1DM vs. T2DM was validated by both QRT-PCR (Fig. 4b) and *in situ* hybridization (Fig. 4c). It was more pronounced in T2DM than in normal limbus and was upregulated significantly in T1DM vs. both T2DM and normal limbi. Further, miR-10b was more expressed in the LESC-harboring basal cell layer in normal limbal epithelium (Fig. 4c) suggesting its roles in stem cell maintenance or early proliferation/differentiation in normal corneal homeostasis. U75 and U6 miRNAs were used as internal and positive controls in QRT-PCR and *in situ* hybridization, respectively, and scrambled sequences were used as negative controls (Figs 3 and 4).

### miR-10b predicted targets and Gene Ontology analysis

Due to significant differential enrichment of miR-10b in the limbus and in diabetic cornea, we further investigated its potential biological function by computational analysis using miRTarBase, the experimentally validated miRNA-target interactions database, to find predicted targets for miR-10b. 294 predicted target genes of miR-10b-5p were extracted from miRTarBase. The predicted target gene list was used to perform the gene ontology (GO) enrichment analysis using DAVID Functional Annotation Bioinformatics Resources v6.8. The significantly enriched GO terms of biological process are shown in Supplementary Table S5. The potential target genes are only presented by selecting epithelium-related genes and the experimentally validated targets. *HOXD10* gene was among the high predicted and experimentally observed miR-10b targets. Interestingly, most of the significant GO enrichments for validated and/or epithelial-related genes were related to regulation of transcription, differentiation, apoptosis, cell cycle and proliferation. It seems that miR-10b target genes are mostly involved in transcription regulation and signal transduction, such as Ras and canonical Wnt signaling pathways, which are both involved in a wide range of cellular processes including gene expression, proliferation, differentiation, and apoptosis.

### Functional analysis of miR-10b in corneal epithelial homeostasis

By deep sequencing, miR-10b was one of the most abundant miRNA in the limbus and had the greatest fold difference in limbus vs. central cornea (91.844 fold, Adjusted p < 1.18E-07). By *in situ* hybridization it was more pronounced in the basal and suprabasal epithelial cell layers (Fig. 4c).

#### miR-10b enhances cell proliferation in human organ-cultured cornea and in cultured cells *in vitro*

**Figure 5:**
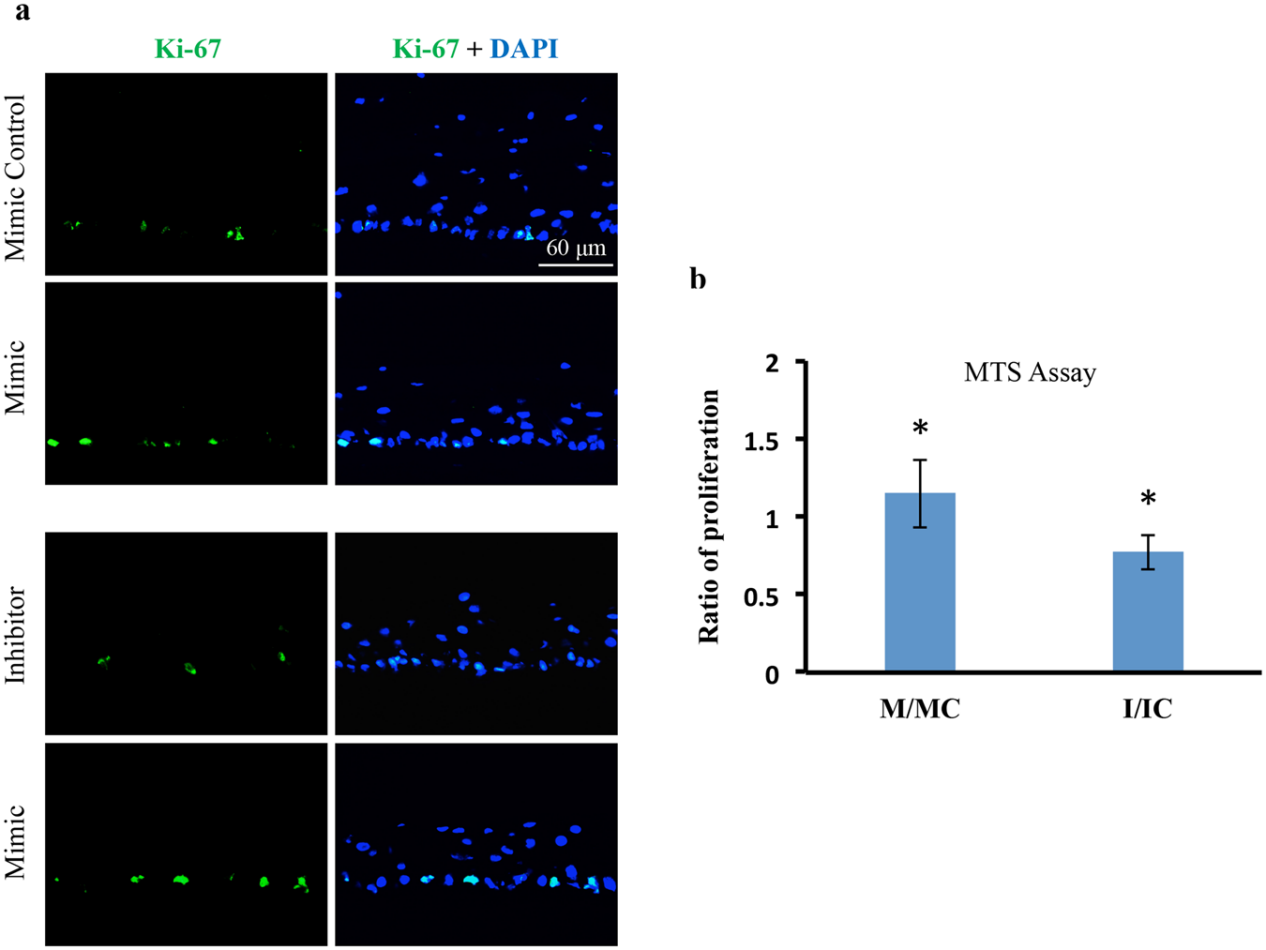
Effect of miR-10b on proliferation in normal human organ-cultured corneas and HCEC. (**a**) miR-10b treatment in human organ-cultured corneas led to increased expression of Ki-67 in mimic-treated cornea compared to the fellow cornea treated with scrambled control, mimic control, or miR-10b inhibitor. (**b**) MTS proliferation assay showed significant increase or decrease in proliferation rates in HCEC transfected with miR-10b (M), or miR-10b inhibitor (I), respectively, in comparison to their respective transfected scrambled controls, mimic control (MC) and inhibitor control (IC). *P < 0.05, compared with scrambled control values by paired two-tailed t test.

First, we verified the increased miR-10b expression levels by QRT-PCR in human organ-cultured cornea, cultured telomerase-immortalized human corneal epithelial cells (HCEC) and primary LEC transfected with miR-10b mimic (M) or its inhibitor in comparison to their corresponding controls (Supplementary Fig. S2a–c). Although present in corneas and HCEC, in primary LEC miR-10 was only detected in isolated cells (passage P0), but not in cells at later passages such as in P1, P2 and P3 (Supplementary Fig. S2d). Human organ-cultured corneas transfected with miR-10b showed increased Ki-67 immunostaining in the epithelial basal cell layer in comparison to the transfected corneas with mimic control or miR-10b inhibitor (Fig. 5a). Further, we assessed the effects of miR-10b overexpression or inhibition on cultured HCEC proliferation rate using MTS cell proliferation assay. Overexpression of miR-10b or its inhibition in cultured HCEC showed increase or decrease in cell proliferation, respectively (Fig. 5b). We also examined the effect of miR-10b on cell migration in HCEC by gain- and loss- of function analyses. Overexpression of miR-10b or its inhibition in HCEC had no significant effect on cell migration rates compared to controls as determined by a scratch wound healing assay.

#### mRNA profiling using Fluidigm QRT-PCR reveals potential targets of miR-10b in HCEC

**Figure 6:**
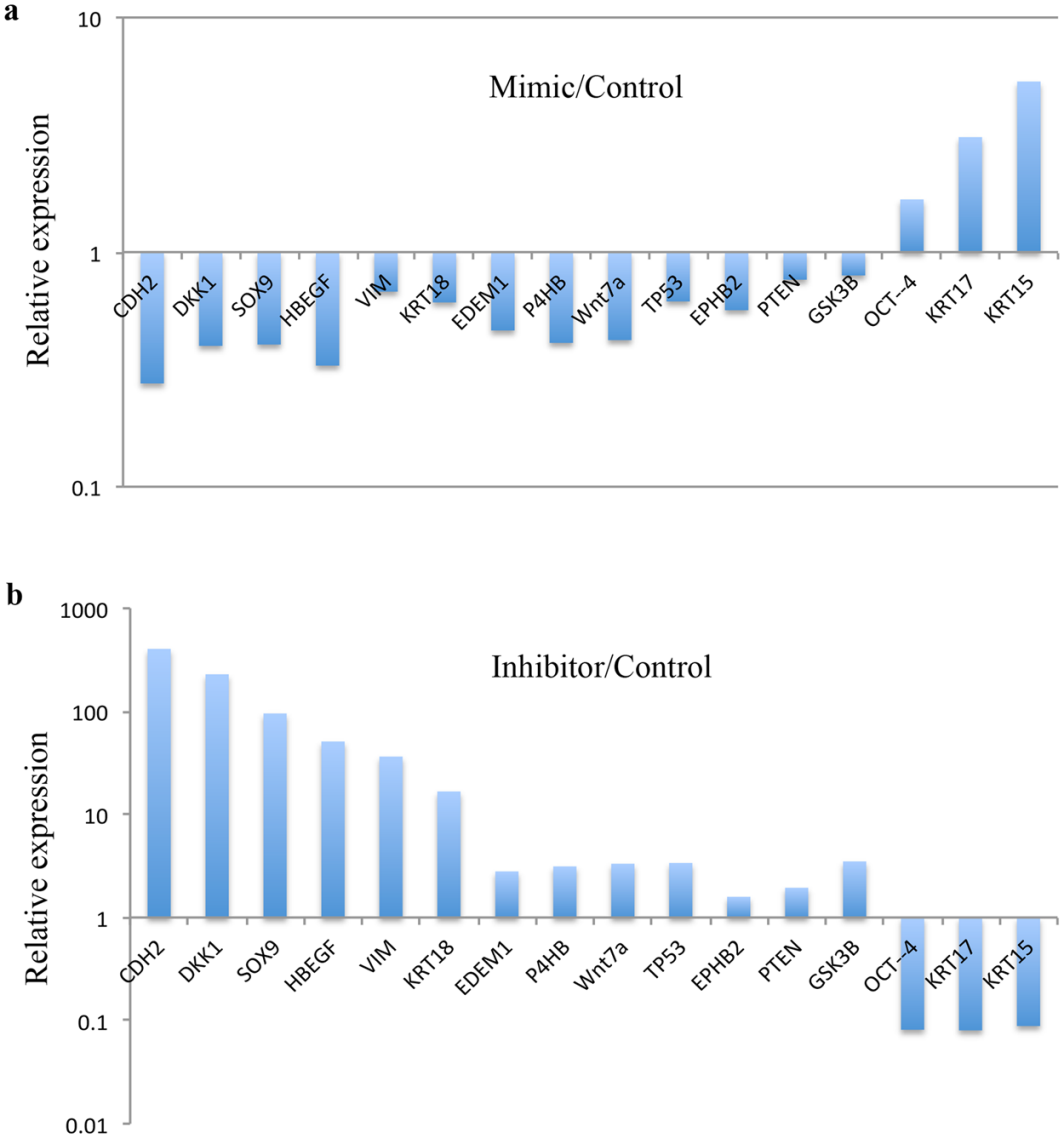
miRNA Profiling of miR-10b Transfected HCEC by Fluidigm QPCR. Transfection with miR-10b mimic using RNAiMax decreased significantly the mRNA expression level of some putative LESC markers and miR-10b predicted targets (**a**). Transfection with miR-10b inhibitor significantly increased their expression levels (**b**) in HCEC compared to their corresponding scrambled sequence controls (mimic control and inhibitor control, respectively). Further, the putative LESC marker mRNA levels that were increased in HCEC transfected with miR-10b, showed decreased levels in HCEC transfected with miR-10b inhibitor in comparison to corresponding controls. Student’s t-test P < 0.05.

We performed the mRNA profiling of HCEC with both miR-10b up regulation and down regulation by Fluidigm QRT-PCR using a panel of primers specific to putative stem cell markers and miR-10b predicted targets (Supplementary Table S6). Overexpression of miR-10b in cultured HCEC decreased, whereas its inhibition significantly increased mRNA expression levels of predicted miR-10b target genes *HBEGF*, *EDEM1*, *TP53*, and *PTEN* (Fig. 6a,b) and putative LESC marker genes, *CDH2* and *SOX9*, compared to their corresponding controls. However, two other LESC/limbal epithelial marker genes, *KRT15* and *KRT17*, and pluripotent stem cell marker gene, *OCT4*, showed significant increase with miR-10b overexpression and significant decrease with miR-10b inhibition in HCEC (Fig. 6a,b). LESC marker genes *∆NP63*, *KRT14* and differentiated epithelial cell marker genes *KRT12* and *KRT3* did not show any significant changes in their mRNA expression level in transfected HCEC. Interestingly, mRNA expression levels of Dickkopf Wnt signaling pathway inhibitor 1 (*DKK1*) and glycogen synthase kinase-3β (*GSK-3β*), which is also a Wnt signaling inhibitor, decreased upon miR-10b overexpression, whereas a significant increase in miR-10b inhibitor-transfected HCEC (Fig. 6a,b) was observed. These data suggested a possible activation of Wnt signaling in miR-10b-transfected HCEC.

#### miR-10b regulates K17 in HCEC culture and in human organ-cultured corneas

**Figure 7:**
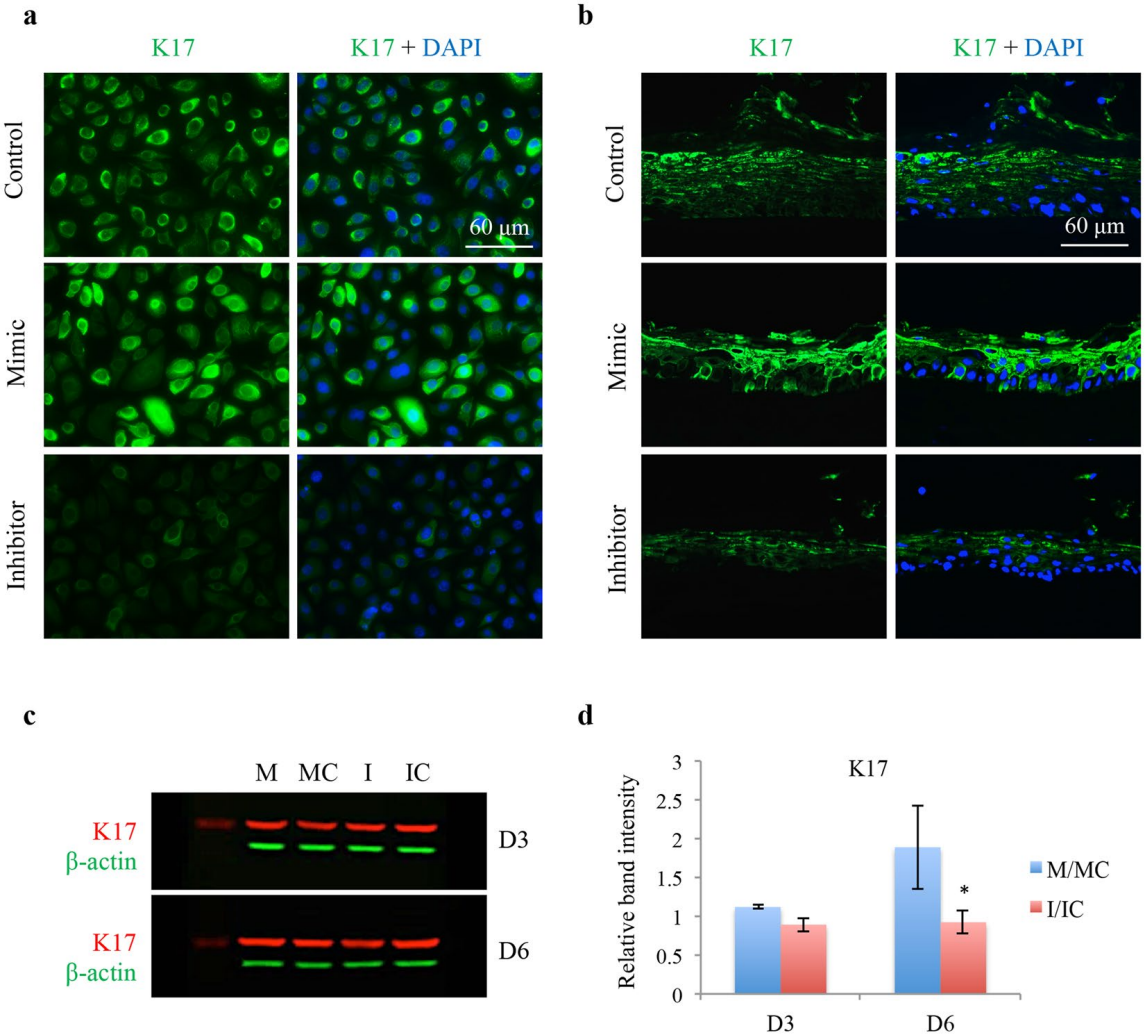
Effect of miR-10b on LEC marker K17 expression in HCEC and in normal human organ-cultured corneas by immunostaining and western blot. (**a**) Transfection with miR-10b increased, whereas with miR-10b inhibitor decreased staining for K17 in HCEC compared to their corresponding controls. (**b**) MiR-10b treatment in human organ-cultured corneas led to increased expression of K17 compared to scrambled mimic control or Inhibitor. The same exposure time was used for each set of compared immunostained sections, and the assessment was done by more than one observer. The pictures are representative of two to three independent experiments. (**c**) Western blot analysis, total extracted protein from transfected HCEC with miR-10b mimic (M) or its inhibitor (I) and their corresponding controls, mimic control (MC) and inhibitor control (IC), respectively, was separated on gradient SDS-PAGE gels, transferred to membrane and probed with K17 antibody (Table 2). Antibody to β-actin was used as loading controls and for semi-quantitation. miR-10b treatment decreased, whereas its inhibitor increased, protein level of K17. (**d**) Quantitation of K17 protein level. The bar graph represents average ± SEM of pooled values (n = 4) of densitometric scans. *P < 0.05, compared with scrambled control values by paired two-tailed t test.

Overexpression of miR-10b in cultured HCEC increased the expression of keratin 17 (K17) at the protein level, and the inhibitor had an opposite effect (Fig. 7a). Additionally, immunostaining of organ-cultured corneas treated with miR-10b mimic showed a similar increase in K17 compared to the fellow corneas transfected with scrambled sequences, mimic control, or miR-10b inhibitor (Fig. 7b). Further, miR-10b treatment of HCEC led to modest increase in K17 expression vs. scrambled mimic control, whereas silencing of miR-10b using its inhibitor led to decreased K17 expression by western blot (Fig. 7c,d).

#### Verification of miR-10b predicted targets in cultured HCEC and human organ-cultured corneas

**Figure 8:**
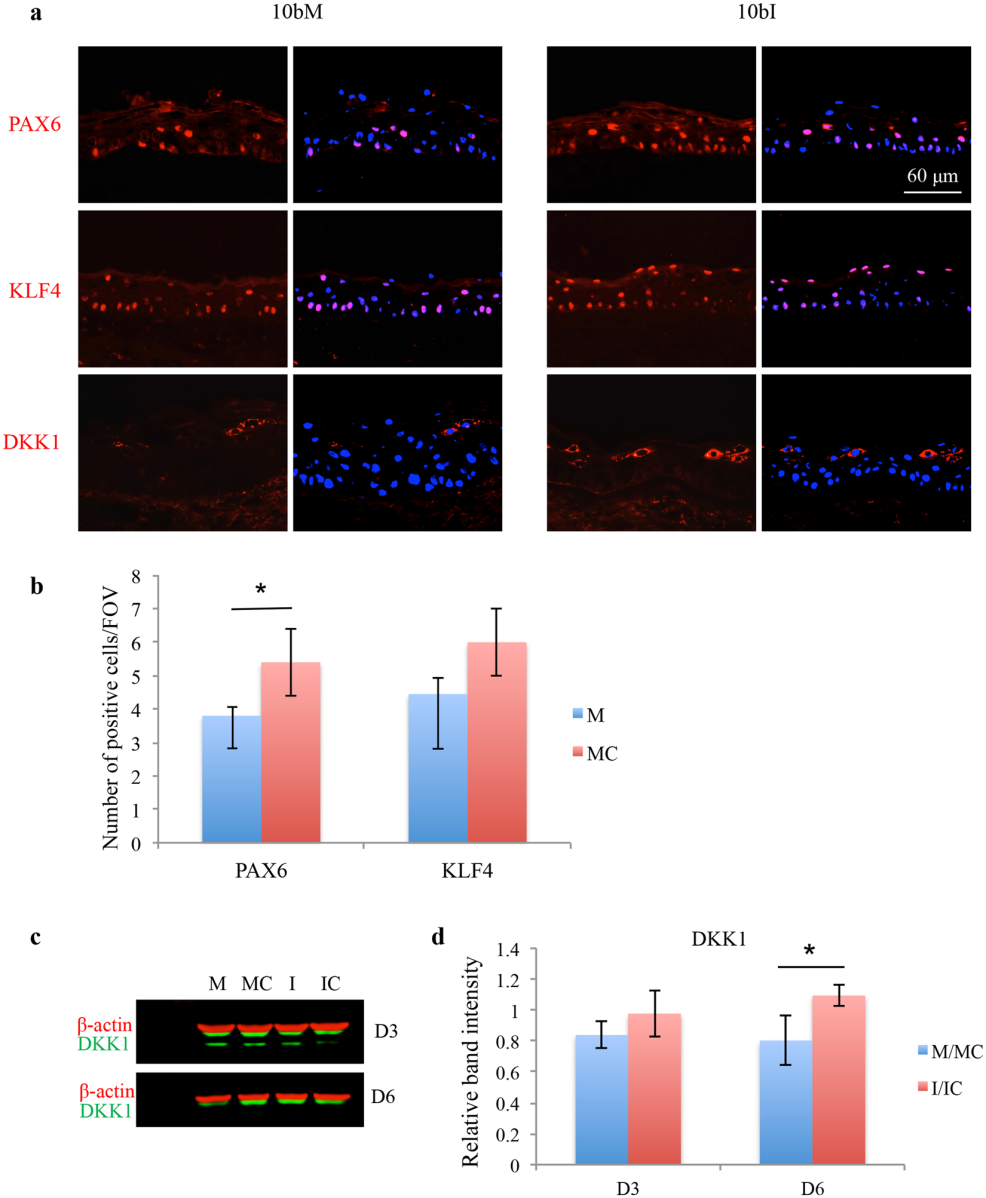
Effect of miR-10b on its predicted targets in human organ-cultured corneas and in HCEC. (**a**) Transfection with miR-10b (10bM) showed decreased, whereas with miR-10b inhibitor (10bI) increased staining for PAX6, KLF4 and DKK1 in human organ-cultured corneas (n = 4). The same exposure time was used for each set of compared immunostained sections, the pictures are representative of two to three independent experiments of each transfected organ-cultured corneas (n = 4). (**b**) The staining assessment was done by more than one observer. The bar graph represents average ± SEM of pooled values (n = 4) in duplicates or triplicates. (**c**) Western blot analysis of total extracted protein from transfected HCEC with miR-10b mimic (M) or its inhibitor (I) and their corresponding controls, mimic control (MC) and inhibitor control (IC), respectively, after day 3 (D3) or day 6 (D6), and (**d**) Quantitation of DKK1 protein level in western blot. The bar graph represents average ± SEM of pooled values (n = 4) of densitometric scans. *P < 0.05, compared with scrambled control values by paired two-tailed t test.

*In silico* analysis of miR-10b predicted targets using several target prediction software programs along with our mRNA profiling revealed potential target proteins that are known to regulate corneal epithelial homeostasis (Table S5 and Fig. 6), such as PAX6, KLF4, and DKK1. Immunostaining for two potential targets, PAX6 and KLF4, in miR-10b transfected organ-cultured corneas revealed significant down-regulation of PAX6 vs. mimic control or inhibitor-transfected organ-cultured corneas; while, KLF4 expression had a tendency toward down regulation that did not reach significance (Fig. 8a,b). However, in cultured HCECs, changes observed in KLF4 or PAX6 expression level upon miR-10b overexpression or inhibition were non-significant. Interestingly, DKK1 (Wnt signaling inhibitor) expression level decreased upon miR-10b overexpression both in organ-cultured corneas by immunostaining and in cultured cells by western blot, with an opposite effect observed upon miR-10b inhibition (Fig. 8a–c). The difference was statistically significant after 6 days of treatment.

## Discussion

The present study revealed differential expression of specific miRNAs in anatomically and functionally distinct corneal compartments in both healthy and diabetic corneas. Interestingly, in the limbus, which is more metabolically active than central cornea, most miRNAs had much higher expressions suggesting their possible roles in LESC maintenance and differentiation both in normal and diseased tissue. Using deep sequencing with QRT-PCR validation, we have identified a number of differentially expressed miRNAs in limbus vs. central cornea, some of which have not been documented previously (Table S1). In fact, miRNA expression profile of human diabetic corneas has been studied here for the first time using deep sequencing. Although there are some common overexpressed or underexpressed limbal vs. central miRNAs in normal and diabetic corneas, there are some unique differences in the diabetic limbus (Fig. 1e) suggesting altered expression contributing to the disease state. Furthermore, we assessed for the first time the miRNAs that are differentially expressed in human diabetic vs. normal limbus, which were also validated by QRT-PCR. The difference in T1DM and T2DM miRNA expression profiles in the limbal cornea was a striking and novel observation as confirmed by several analyses (Figs 1f and 2a,b).

The miRNA expression profiles in normal human cornea revealed miR-10b as one of the most abundant miRNAs in the limbus. Moreover, as documented by *in situ* hybridization and a previous study^24^, miR-10b expression was more pronounced in the basal cell layers. This finding suggests its possible role in stem cell maintenance or early TA cell differentiation. Additionally, we confirmed its upregulation in diabetic vs. normal limbus with higher expression in T1DM vs. T2DM, where it was expressed in basal, suprabasal, and superficial epithelial layers suggesting that its different spatial expression compared to normal limbus may contribute to diabetic corneal disease. Interestingly, miR-10b expression, which was identified in immortalized cultured HCEC, *ex-vivo* and organ-cultured corneas, was only detected in isolated primary LEC at P0, but not in subsequent culture cells at later passages^24^ (Supplementary Fig. S2d). This suggests that miR-10b is involved in normal LEC maintenance *in vivo* and calls for careful interpretation of cell culture data. miR-10b has been shown to be an important cell regulator of a wide variety of cell functions, and can target different genes in different cell types. miR-10b is associated with tumor growth and can modulate the epithelial-mesenchymal transition (EMT), which is a critical step toward pluripotency^25–27^. Its possible target in neural cell differentiation is NCOR2^28^, as well as major transcription factors controlling eye development, PAX6, Krüppel-like factor 4 (KLF4) and Notch-1^26, 27^. Although considerable studies have been done on the biological functions and downstream targets of miR-10b in cancer cells, much is still to be revealed on its physiological roles in normal cells. miR-10b can act upon PAX6 and KLF4 that are transcription factors playing important roles in the development and maintenance of the cornea. KLF4 regulates cell cycle and is elevated in terminally differentiated epithelial cells^29–31^. Therefore, downregulation trend of KLF4 in miR-10b transfected human organ-cultured corneas may suggest the role of miR-10b in early proliferation/differentiation of LESC. Increased epithelial proliferation with downregulation of desmosomal components leading to epithelial fragility in the corneas of KLF4 null mice^30^ is consistent with diabetic corneal alterations^32, 33^.

PAX6 has a major role in corneal epithelial homeostasis, differentiation and wound healing^34–37^. In addition, EGF-induced proliferation leads to down-regulation of PAX6 expression for corneal epithelial proliferation^38^. A central role of the Wnt7A–PAX6 axis in corneal epithelial cell fate determination and differentiation has been recently suggested^39^. Interestingly, our mRNA profiling showed down-regulation of Wnt7A in HCEC with overexpressed miR-10b, and its up regulation upon miR-10b inhibition. The observed trend toward down regulation of both Wnt7A and PAX6 in HCEC with overexpressed miR-10b may suggest a possible role of miR-10b in LESC proliferation and/or their differentiation to TA cells. PAX6 mutation is common in abnormal eye development leading to aniridia, glucose intolerance and development of diabetes^40, 41^. PAX6 impaired function or its conditional inactivation causes early onset of diabetes and corneal opacification^42, 43^. Therefore, altered spatial overexpression of miR-10b in diabetic limbus and downregulation of PAX6 in suprabasal and superficial layers may contribute to diabetic corneal alterations^44^.

Keratins have been used as marker proteins at distinct stages during cellular epithelial differentiation^45–47^. K15 is expressed in all types of developing stratified epithelia and is considered as one of the earliest stratification-related keratins^48^. Both K15 and K17 are considered as putative stem cell markers due to their expression in the basal cell layer of normal squamous stratified epithelia^49, 50^; they may also be expressed in TA cells. Additionally, K15 has been shown as a marker of laterally differentiating epidermal keratinocytes in the basal layer^51^. Therefore, significant upregulation of K15 and K17 mRNA levels in miR-10b transfected HCEC may suggest a regulatory role of miR-10b in early and lateral differentiation of LESC to TA cells. New roles of keratins have emerged beside their intracellular structural and mechanical functions^52–54^. These include the role of K17 in cell survival and protection from apoptosis^53^, the regulation of protein synthesis and cell growth during wound healing involving K17 in intracellular signaling pathways^54^, and cell-cycle progression and proliferation signaling^55, 56^.

Downregulation of DKK1 in miR-10b transfected HCEC and organ-cultured corneas along with the downregulation of mRNA level of GSK-3β, a Wnt signaling inhibitor, may suggest a role of miR-10b in activating Wnt signaling in limbal epithelium. Wnt signaling has been implicated in the maintenance and expansion of adult stem cells^57–59^ as well as of human limbal stem/progenitor cells^60^. Maintenance of corneal epithelial homeostasis depends on the precise regulation of LESC function. LESC have to rapidly respond to different cell conditions such as regeneration or tissue damage, proliferate to the exact need and differentiate accordingly to maintain tissue homeostasis. These cellular functions may be regulated by miRNAs, which have the capability of fine-tuning gene expression due to their partial base-pairing and adjustability in translational gene regulation in order to respond to the needs of the cell at any given time^61^. Therefore, the miRNA-induced changes in protein levels are usually modest^61, 62^, which may explain weak miR-10b repression of the K17 and DKK1 levels by western blot in our study. Alternatively, several miRNAs may fine-regulate gene expression cooperatively.

Our study thus suggests that miR-10b regulates LESC early proliferation state during renewal and/or division when cells exit the stem cell niche going through their lateral differentiation to become TA cells. This may involve fine-tuned regulation of proliferation to the exact need of the tissue by miR-10b through its downregulation of DKK1 and GSK-3β, which leads to upregulation of Wnt signaling^24, 57, 60^. Further, miR-10b indirectly regulates LESC/TA cell K17, which may be through direct targeting of p53^63^. Eventually, the committed cells differentiate while migrating laterally and upward out of the reach of Wnt signaling and under PAX6 and KLF4 regulatory mechanisms for terminal differentiation^29–31, 40, 42^. This is compatible with up-regulation of miR-10 in diabetic corneas leading to down-regulation of PAX6 resulting in delayed wound healing, fragility and corneal opacification, characteristics of diabetic cornea^42^.

In summary, we used microRNA deep sequencing in order to advance our understanding of the role of miRNAs in normal and diseased corneal states. We identified miRNA expression profiles in human normal and diabetic limbus, and found a number of miRNAs that were differentially expressed between normal and both T1DM and T2DM corneas. For the first time, we have found a subset of miRNAs that are differentially expressed between T1DM vs. T2DM, further suggesting that Type 1 and Type 2 diabetes are different diseases though having the same outcome and complications. Physiological significance of this finding warrants further investigation.

The examined miRNAs, especially miR-10b that is one of the most abundant limbal miRNAs, may regulate corneal epithelial homeostasis and stem cell functions. They may be considered as excellent candidates for functional studies of limbal progenitor cells in normal and disease states and as possible new tools for treating diabetic keratopathy.

## Materials and Methods

### Human tissues

Age-matched human autopsy normal and diabetic cadaver corneas (Table 1) were received from the National Disease Research Interchange (NDRI, Philadelphia, PA) in Optisol storage medium (Chiron Vision, Claremont, CA; donor identity was withheld by the supplier. NDRI has a human tissue collection protocol approved by a managerial committee and subject to National Institutes of Health oversight. In all cases the required informed consent from donors’ next of kin specifying the use of postmortem tissue for research was obtained by NDRI-affiliated eye banks. The work reported here was covered by approved IRB protocols EX-1055 and Pro00019393 from Cedars-Sinai Medical Center. Corneas/globes were harvested within 5 hours of donor death and reached our laboratory within 24 hours of donor death, and studies were conducted in accordance with approved guidelines.

**Table 1:** List of corneas used in this study.

Case number	Age	Gender	Cause of death	Type of DM	DM duration (Years)	History of eye diseases
N10-21	79	M	Cerebrovascular Accident	N/A	N/A	Cataracts
N10-22	75	F	Coronary artery Disease	N/A	N/A	Cataracts
N10-23	75	M	Cerebrovascular Accident	N/A	N/A	None
N10-24	66	M	Cardiopulmonary Arrest	N/A	N/A	None
N11-11	62	F	Congestive Heart Failure	N/A	N/A	None
N11-27	34	F	Unknown	N/A	N/A	None
N12-01	82	M	Severe Hyponatremia	N/A	N/A	None
N12-03	68	F	Intracerebral Hemorrhage	N/A	N/A	None
N12-08	60	M	Liver Cancer	N/A	N/A	Cataracts
N12-09	74	F	Cancer	N/A	N/A	None
N13-02	57	F	Hepatic Encephalopathy	N/A	N/A	None
N13-03	63	F	Respiratory Failure	N/A	N/A	None
N13-10	82	F	Cardiopulmonary Arrest	N/A	N/A	IOLs OU
N13-45	77	F	Respiratory Arrest	N/A	N/A	None
N14-03	91	F	Cerebrovascular Accident	N/A	N/A	None
N14-14	80	M	Acute Cardiac Event	N/A	N/A	None
N14-37	90	M	Respiratory Failure	N/A	N/A	None
N16-33	79	F	Respiratory Failure	N/A	N/A	IOLs OU
N16-35	76	F	Stroke/CVA	N/A	N/A	None
DM11-18	70	F	Cerebrovascular Accident	NIDDM	20	None
DM11-30	39	M	Renal Disease	IDDM	Unknown	Hematoma behind retina
DM11-31	69	M	Renal Failure	IDDM	Unknown	None
DM12-02	74	F	Renal Failure	NIDDM	10–15	Cataracts OU
DM12-05	51	M	Ventricular Fibrillation	IDDM	Unknown	None
DM12-06	56	F	Anoxic Encephalopathy	NIDDM	15	IOLs OU
DM12-07	46	M	Ischemic Heart Disease	NIDDM	7	None
DM12-10	74	M	Anoxic Encephalopathy	NIDDM	15	None
DM12-11	88	M	Hypertension	NIDDM	20	IOLs OU
DM12-12	60	M	CHF	IDDM	5	None
DM12-13	81	M	Cardiac/Respiratory Arrest	IDDM	6	None
DM12-14	80	F	Respiratory Failure	IDDM	15	None
DM13-01	78	F	Pulmonary Disease	NIDDM	Unknown	None
DM13-02	91	M	Pneumonia	NIDDM	6	IOLs OU
DM13-03	63	F	Respiratory Failure	IDDM	Unknown	None
DR13-11	53	F	Respiratory Failure	IDDM	20	IOLs OU
DM13-13	78	M	Respiratory Failure	NIDDM	31	None
DM14-12	80	F	Stroke	IDDM	45	IOLs OU
DM14-50	68	M	Respiratory Failure	IDDM	28	IOLs OU

N, normal; DM, diabetic mellitus; IDDM, T1DM; NIDDM, T2DM; M, male; F, female; IOL, intraocular lens; OU, both eyes.

### Total RNA isolation

Total RNA including low molecular weight (LMW) RNA was extracted using mirVana miRNA kit (Thermo Fisher Scientific, Waltham, MA) from age-matched normal (n = 10) and DM (n = 12) human corneas that were 8.5 mm-trephined into central and limbal components according to the manufacturer’s instructions. RNA concentration and quality were assessed using NanoDrop 8000 spectrophotometer (Thermo Scientific, Waltham, MA), Qubit 2.0 fluorometer (Invitrogen/Life Technologies, Waltham, MA) and Agilent 2100 bioanalyzer system (Agilent Technologies, Santa Clara, CA).

### Next-Generation miRNA sequencing (miRNA deep sequencing)

#### Sample preparation and sequencing

One µg of total RNA was used for sequencing library preparation from 10 normal and 12 diabetic corneas both central and limbal compartments, a total of 44 samples, with Illumina TruSeq™ Small RNA Library Prep kit per the manufacturer’s protocol. In brief, adapters were ligated to both ends of the RNA, transcripts were reverse transcribed, and then cDNA was PCR amplified with indexed primers to enable multiplexing samples for sequencing. Libraries between 145–160 base pairs long were purified from gel. Libraries were pooled in equimolar concentration for each experiment (8 per lane), and clustered on a single-end flow cell using an Illumina c-bot. The library-clustered flow cell was sequenced on the Illumina GAIIx, 1 × 36 bp sequencing chemistry.

### Bioinformatics analysis

#### Quality control and alignments

Fastq files were extracted de-multiplexed using Illumina’s Casava V8.1 package. First low-quality reads from the original sequencing data were removed by Trim Galore^64^. Adaptor sequences were removed using a gapped local alignment (Smith-Waterman alignment; Match:10, Mismatch: −5, Indel: −8) using a 5 bp match. High quality reads of ~30 bp were retained. Quality reads were aligned to human genome GRCH 37 using Bowtie-1 allowing only two mismatches and best stratum alignment options. Annotation was obtained from various ncRNA databases and Gencode v19 (human genome) and mirBase 20 were used. Exon and intron boundaries were marked and coding regions were excluded. Reads were considered to be miRNAs they possessed 100% identity at the sequence level and covered the whole length of the miRNA based on the sequences in miRBase. Reads were quantified as sequence tags in each library separately and normalized as reads per million (RPM) essentially as advocated in previously published literature^65, 66^, except that we additionally used a local alignment to remove the adaptor sequences.

#### miRNA filter and differential expression

A miRNA with no read count in all samples were excluded. Further miRNAs that showed read count >10 in 75% of samples were retained for further global analysis except that for comparison of diabetic vs. normal and T1DM vs. T2DM two group analysis miRNAs showing read counts >10 reads in all the samples were considered. Differential expression in two groups comparison was calculated using one way ANOVA/t-test. A FDR controlled p value was computed using Benjamini and Hochberg procedure for multiple hypothesis testing. To further rank differentially expressed miRNAs, the data was shifted to log2 values and fold change was calculated.

### Quantitative Real-Time RT-PCR (QRT-PCR) and *in situ* hybridization

QRT-PCR was performed as described previously^14^. Briefly, 10 ng of total RNA were reverse transcribed (RT) using Taqman MicroRNA RT kits and miRNA sequence-specific primers (Thermo Fisher Scientific). QPCR was carried out using Taqman 2X universal PCR Master Mix (no AmpErase UNG) along with Taqman 20X MicroRNA Assays (Thermo Fisher Scientific). Each well contained 1.33 µl of RT reaction product, 1X Taqman PCR Master Mix, and 1X specific miRNA primer, designed to detect mature miRNAs. Amplification was carried out on the ViiA^TM^ 7 Real Time PCR System (Thermo Fisher Scientific). Each sample was run in triplicate. Signals were normalized to small nucleolar RNA U75 (SNORDU75). U75 housekeeping miRNA run in parallel. A comparative threshold cycle (Ct) method (ΔΔCt) was used to calculate relative miRNA expression. High-throughput QRT-PCR on a custom gene panel was performed on a Fluidigm BioMark HD system in the Genomic Core at Cedars-Sinai Medical Center per manufacture instruction. Primers for miR-10b predicted target and putative LESC and corneal epithelial marker genes are listed in Supplementary Table S6. *In situ* hybridization was performed as described previously^12^.﻿

### Isolation and culture of primary limbal epithelial cells

Primary corneal limbal epithelial cell (LEC) cultures were established as described previously^14^. Briefly, epithelial cells from corneoscleral rims were isolated by Dispase/Trypsin digestion and grown in Epilife containing Human Corneal Growth Supplement (HCGS), N-2 Supplement, B27 Supplement, 1X antibiotic/antimycotic mixture, plus 10 ng/mL epidermal growth factor (EGF) (Thermo Fisher Scientific). In addition, primary LEC were maintained in 10 µM Rho-associated protein kinase (ROCK) inhibitor (Stemgent, Cambridge, MA) in P0 and removed prior to passage for experiments.

### Transfection of human primary limbal epithelial cells and organ-cultured corneas

Human Corneal Epithelial Cells (HCECs)^12^ were transfected with 50 nM hsa-miR-10b-5p mimic (miRCURY LNA™ microRNA mimic, Exiqon) or inhibitor (miRCURY LNA™ microRNA inhibitor, Exiqon) or respective negative controls using Lipofectamine RNAiMAX transfection reagent (Thermo Fisher Scientific) following the manufacturer’s instructions. At Day 3 and 6 post-transfection, cells were either harvested for western blotting, RNA isolation or processed for immunocytochemistry or functional assays.

Corneal organ cultures were established as described^50^ and were maintained in Dulbecco’s Modified Eagle’s Medium (Thermo Fisher Scientific) with 1X insulin-transferrin-selenite (Sigma-Aldrich), 1X non-essential amino acids, and 1X antibiotic/antimycotic mix (Thermo Fisher Scientific). Each cornea in a pair was transfected with 50 nm hsa-miR-10b mimic or miR-10b inhibitor, with respective negative controls for 48 hours using Lipofectamine RNAiMAX transfection reagent. After additional incubation period of 3–5 days, the transfected corneas were processed for immunohistochemistry.

### *In vitro* scratch wound assay

HCEC were seeded on coated 12-well plate and transfected with 10b mimic, inhibitor or respective controls as described earlier. Transfected HCEC at confluence were scratch wounded using the pipette tip and photographed at time 0. The wounds were allowed to heal and photographed every 6 h. All images were then analyzed using ImageJ software. The percent area healed was calculated with reference to time 0.

### Cell proliferation (MTS) assay

To assess the effect of miR-10b on proliferation of HCECs, cells were seeded on 96-well plate at 5000/well and transfected either with miR-10b mimic, or inhibitor or their respective negative controls at final concentration of 50 nM. At the study time points (day 3 and 6 post-transfection), proliferation was measured using MTS assay (CellTiter 96 Aqueous One Solution Cell Proliferation Assay). Briefly, 20 μl of MTS reagent was added to 100 μl culture media and incubated in cell culture incubator for 4 h. At the end of the 4 h incubation, the color change due to reduction of formazan by live proliferating cells was measured with a micro-plate reader by absorbance at 490 nM.

### Immunostaining

Cultured HCEC or 5-µm thick corneal transverse cryostat sections were fixed in 4% paraformaldehyde (PFA) and 1% formalin, respectively. Then, cultured cells were permeabilized in 0.2% Triton X-100 for 10 min at room temperature, then blocked for 1 h in a 2% bovine serum albumin (BSA) in PBS solution. Both cultured cells and corneal tissues were incubated with primary antibodies (Table 2) in blocking solution overnight at 4 °C. It followed the next day by1 hr incubation of cross-species adsorbed secondary antibodies conjugated with either fluorescein isothicyanate (FITC) or tetramethylrhodamine (TRITC) (Jackson ImmunoReseach Laboratories, West Grove, PA) the dark at room temperature. For each marker the same exposure time was used when photographing stained sections. The staining was assessed using six exclusive fields of view for each biological replicate for quantitative analysis using ImageJ software. Briefly, images were converted to 8-bit grayscale and after background subtraction each image was set to a predetermined threshold for pixel size and then particles were analyzed. In order to count smudged cells as individual cells, watershed function was used. At least two independent blinded observers performed the analysis using same preset parameters. The pictures are representative of two to three independent experiments. Negative controls without a primary antibody were included in each experiment.

**Table 2:** List of antibodies used in this study.

Antigen	Antibody	Source	MW (kDa)	Dilution
PAX6	Rabbit pAb 901301	Biolegend	47–50	1:100
KLF4	Rabbit mAb ab151733	Abcam	54	1:50
K17	Mouse mAb sc-58726	Santa Cruz Biotechnology	46	1:100
β-Actin	Mouse mAb A5316	Sigma-Aldrich	42	1:6000
β-Actin	Rabbit mAb 8457	Cell Signaling Technology	45	1:1000
DKK1	Rabbit mAb ab109416	Abcam	38	1:1000
β-Tubulin	Rabbit mAb 2128	Cell Signaling Technology	55	1:1000
Ki-67	Rabbit mAb ab92742	Abcam	359	1:200

pAb, polyclonal antibody; mAb, monoclonal antibody.

### Western blot analysis

Western blotting was performed as described previously^14^ with some modifications. Briefly, 8% to 16% gradient Tris-glycine SDS polyacrylamide gels were used (Thermo Fisher Scientific). β-actin (AC-740) or β-tubulin (TUB 2.1) antibodies (Sigma-Aldrich) were used to normalize for gel loading. The transferred nitrocellulose membranes were blocked and incubated with primary antibodies for different predicted targets/proteins of miR-10b (Table 2). IRDye 800CW or 680RD goat anti-mouse or anti-rabbit (Li-Cor Biosciences, Lincoln, NE) was used as secondary antibodies. The blots were scanned using Odyssey CLX imaging system (Li-Cor Biosciences).

### Statistical analysis

Experiments were analyzed by Student’s t-test for two groups, or ANOVA for three or more groups with p < 0.05 considered significant, using Prism6 (GraphPad Software, San Diego, CA). For QRT-PCR analysis, the comparative gene expression is normalized with the housekeeping genes. Fold changes between two clinical conditions are measured with the ΔΔCt method (2^−ΔΔCt^). Genes are selected with both fold changes and p-values from Student’s t-test.

## Electronic supplementary material

Supplementary Figures

Supplementary Tables
