# Supplementary material for: Genome-wide analysis suggests a differential microRNA signature associated with normal and diabetic human corneal limbus
Source: Sci Rep. 2017 Jun 14;7:3448. doi: 10.1038/s41598-017-03449-7 (PMC5471258; doi:10.1038/s41598-017-03449-7)
Supplement: Supplementary file 1 — Supplementary Figures [file 41598_2017_3449_MOESM1_ESM.pdf]

# Genome-wide analysis suggests a differential microRNA signature associated with normal and diabetic human corneal limbus

Mangesh Kulkarni[1,2], Aleksandra Leszczynska[1,2], Gabbie Wei[1,2], Michael A. Winkler[1,2], Jie Tang[3], Vincent A. Funari[3], Nan Deng[4], Zhenqiu Liu[4], Vasu Punj[5], Sophie X. Deng[6], Alexander V. Ljubimov[1,2,6], Mehrnoosh Saghizadeh*[1,2,6]

[1]Biomedical Sciences, [2]Regenerative Medicine Institute Eye Program, [3]Genomics Core, [4]Samuel Oschin Comprehensive Cancer Institute, Cedars-Sinai Medical Center; [5]Keck School of Medicine, University of Southern California, [6]David Geffen School of Medicine, University of California Los Angeles, Los Angeles, California, USA.

- Correspondence to: Dr. Mehrnoosh Saghizadeh, Eye Program, Regenerative Medicine Institute, Cedars-Sinai Medical Center, 8700 Beverly Boulevard, AHSP-A8109, Los Angeles, CA 90048, USA. Tel. 1-310-248-8696, e-mail ghiamm@cshs.org

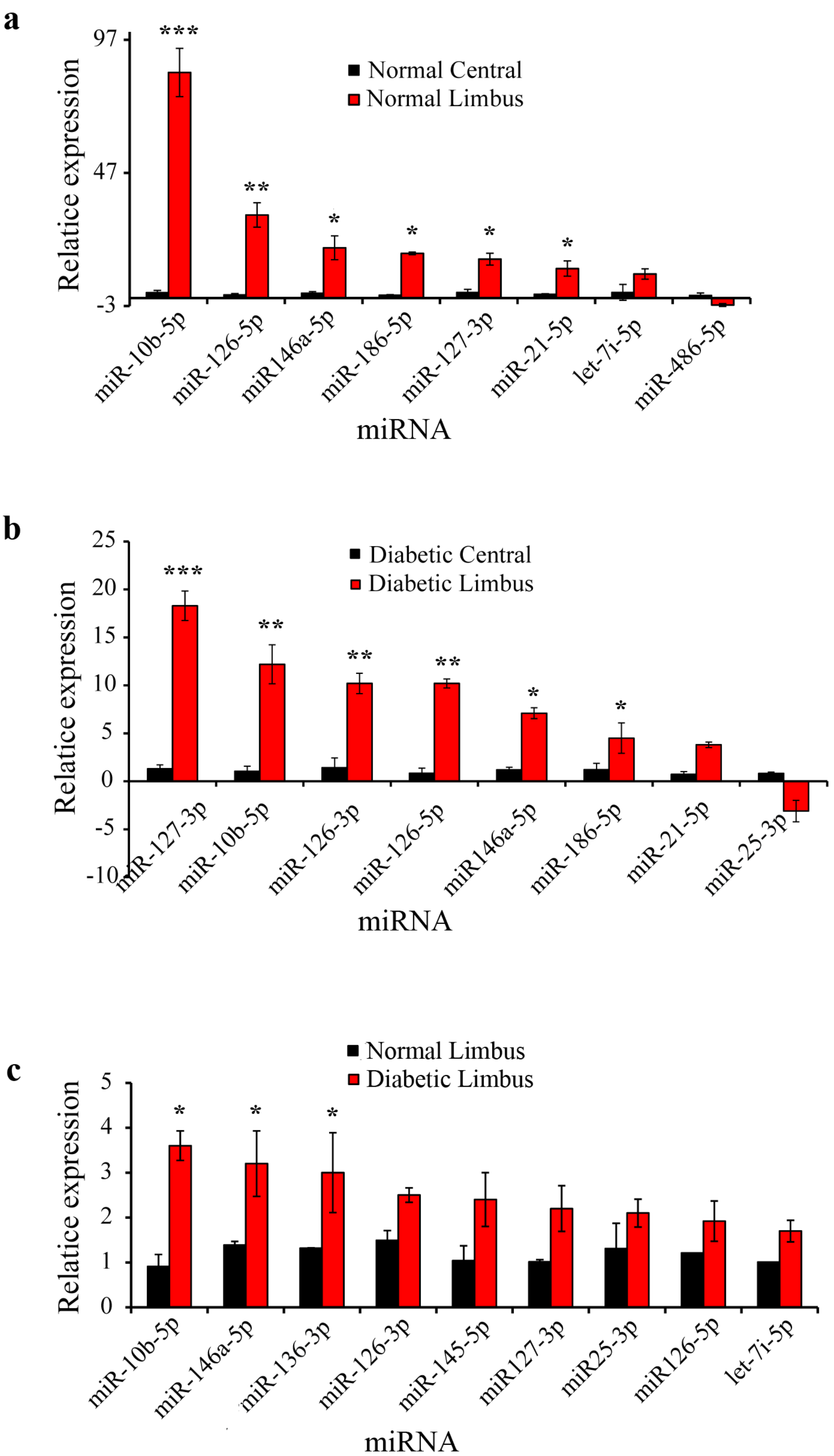


**Supplementary Figure S1. QRT-PCR validation of differentially expressed miRNAs.** Validation of differentially expressed miRNAs in ex vivo central cornea and limbus by QRT-PCR **(a)** Normal limbus vs. central cornea ; **(b)** Diabetic limbus vs. central cornea; **(c)** Diabetic vs. Normal limbus. Bars represent SEM of pooled values (n = 4). Each sample was run in triplicate. Signals were normalized to the U75 housekeeping miRNA run in parallel. A comparative threshold cycle (Ct) method (ΔΔCt) was used to calculate relative miRNA expression. *** $p<0.001$, ** $p<0.01$ and * $p<0.05$ by paired two-tailed t test.

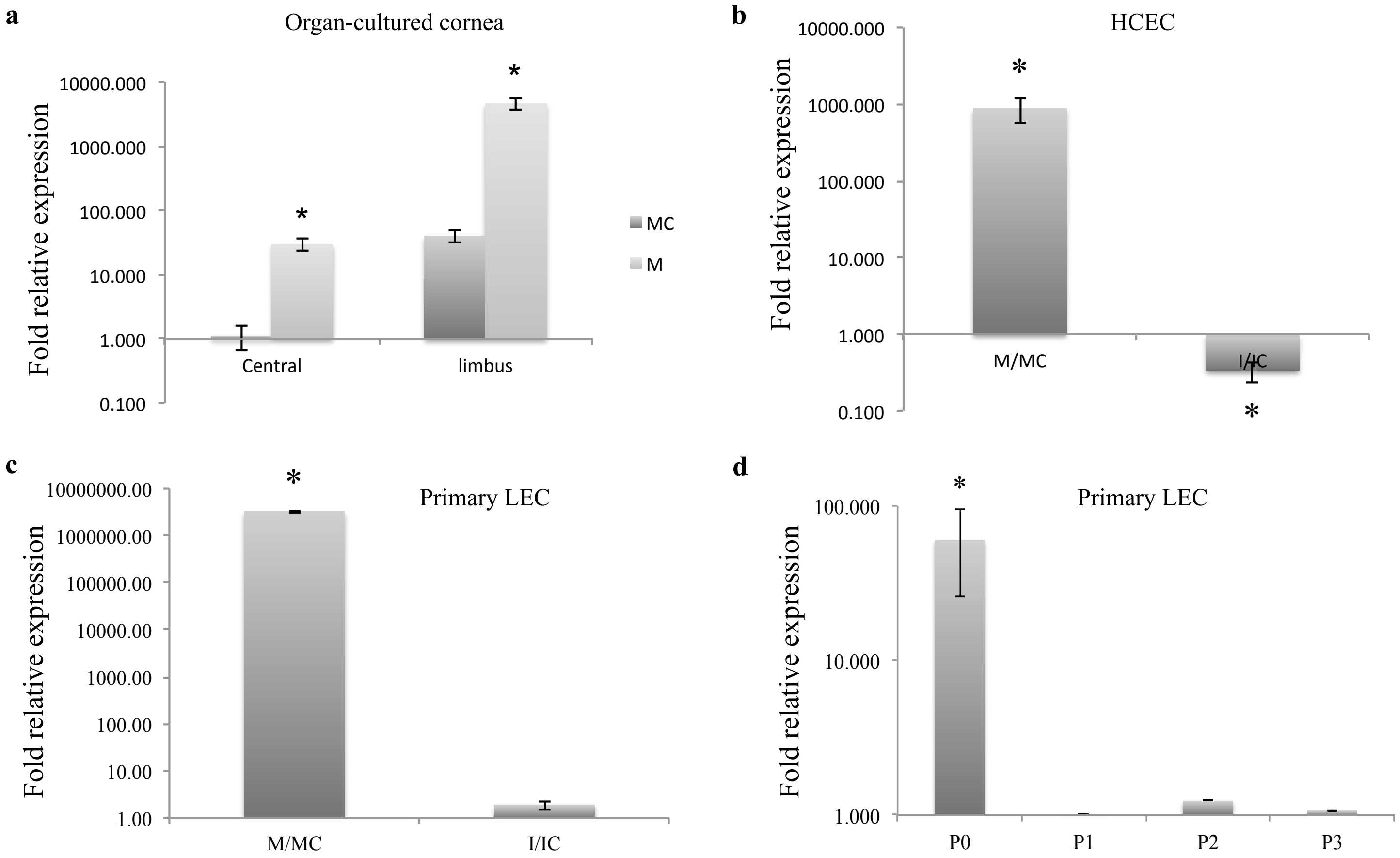


**Supplementary Figure S2. QRT-PCR**. (**a**) miR-10b (M) significant overexpression in transfected central and limbal human organ-cultured cornea in comparison to corresponding mimic control (MC). **(b)** miR-10b significant increase or decrease in HCEC transfected with miR-10b mimic (M) or its inhibitor (I), respectively, in comparison to their corresponding controls, MC or IC. ( **c**) miR-10b significant increase in primary LEC transfected with miR-10b mimic (M) in comparison to mimic control (MC), with no expression observed in MC, I or IC. **(d)** miR-10b significant expression in isolated primary LEC, not yet cultured at passage zero (P0), but no expression was observed in later passages, P1, P2 and P3 LEC.
