# Supplementary material for: Genome-wide analysis suggests a differential microRNA signature associated with normal and diabetic human corneal limbus
Source: Sci Rep. 2017 Jun 14;7:3448. doi: 10.1038/s41598-017-03449-7 (PMC5471258; doi:10.1038/s41598-017-03449-7)
Supplement: Supplementary file 2 — Supplementary Tables [file 41598_2017_3449_MOESM2_ESM.pdf]

Genome-wide analysis suggests a differential microRNA signature associated with normal and diabetic human corneal limbus

Mangesh Kulkarni[1,2], Aleksandra Leszczynska[1,2], Gabbie Wei[1,2], Michael A. Winkler[1,2], Jie Tang[3], Vincent A. Funari[3], Nan Deng[4], Zhenqiu Liu[4], Vasu Punj[5], Sophie X. Deng[6], Alexander V. Ljubimov[1,2,6], Mehrnoosh Saghizadeh*[1,2,6]

[1]Biomedical Sciences, [2]Regenerative Medicine Institute Eye Program, [3]Genomics Core, [4]Samuel Oschin Comprehensive Cancer Institute, Cedars-Sinai Medical Center; [5]Keck School of Medicine, University of Southern California, [6]David Geffen School of Medicine, University of California Los Angeles, Los Angeles, California, USA.

- Correspondence to: Dr. Mehrnoosh Saghizadeh, Eye Program, Regenerative Medicine Institute, Cedars-Sinai Medical Center, 8700 Beverly Boulevard, AHSP-A8109, Los Angeles, CA 90048, USA. Tel. 1-310-248-8696, e-mail ghiamm@cshs.org

Supplementary Table S1. Differentially regulated microRNA expression in normal limbus vs. central cornea (FDR adjusted p-value <0.05)

ID	miRBase accession number	p-value	FDR-adjusted p-value	Fold-Change
**hsa-miR-143-3p**	MIMAT0000435	9.53E-10	1.18E-07	43.02
**hsa-miR-10b-5p**	MIMAT0000254	1.11E-08	6.89E-07	91.84
**hsa-miR-10a-5p**	MIMAT0000253	5.98E-07	2.47E-05	10.57
**hsa-miR-184**	MIMAT0000454	9.56E-06	0.0003	-1.91
**hsa-miR-181a-5p**	MIMAT0000256	1.66E-05	0.0004	-1.63
**hsa-miR-126-5p**	MIMAT0000444	0.0001	0.0022	89.42
**hsa-miR-99a-5p**	MIMAT0000097	0.0001	0.0023	1.85
**hsa-miR-186-5p**	MIMAT0000456	0.0001	0.0023	1.90
**hsa-miR-127-3p**	MIMAT0000446	0.0003	0.0037	62.28
**hsa-miR-199b-3p**	MIMAT0004563	0.0003	0.0042	2.16
**hsa-miR-146b-5p**	MIMAT0002809	0.0005	0.0046	2.44
**hsa-miR-21-5p**	MIMAT0000076	0.0004	0.0046	3.52
**hsa-miR-211-5p**	MIMAT0000268	0.0005	0.0046	5.97
**hsa-miR-146a-5p**	MIMAT0000449	0.0006	0.0052	70.20
**hsa-miR-92a-3p**	MIMAT0000092	0.0010	0.0079	1.58
**hsa-let-7i-5p**	MIMAT0000415	0.0012	0.0095	2.07
**hsa-miR-30a-5p**	MIMAT0000087	0.0014	0.0099	1.65
**hsa-miR-199a-3p**	MIMAT0000232	0.0014	0.0099	2.31
**hsa-miR-27b-3p**	MIMAT0000419	0.0016	0.0103	1.84
**hsa-miR-486-5p**	MIMAT0002177	0.0019	0.0117	-2.66
**hsa-miR-26a-5p**	MIMAT0000082	0.0021	0.0124	1.77
**hsa-miR-23b-3p**	MIMAT0000418	0.0024	0.0138	1.63
**hsa-miR-30d-5p**	MIMAT0000245	0.0037	0.0198	1.40
**hsa-miR-342-3p**	MIMAT0000753	0.0048	0.0250	1.71
**hsa-let-7c-5p**	MIMAT0000064	0.0080	0.0397	1.58
**hsa-miR-26b-5p**	MIMAT0000083	0.0086	0.0409	2.35
**hsa-miR-378a-3p**	MIMAT0000732	0.0097	0.0440	-1.55
**hsa-miR-181b-5p**	MIMAT0000257	0.0099	0.0440	-1.36
**hsa-miR-151a-3p**	MIMAT0000757	0.0112	0.0465	1.48
**hsa-miR-501-3p**	MIMAT0004774	0.0110	0.0465	1.91
**hsa-miR-199a-5p**	MIMAT0000231	0.0126	0.0505	1.95
**hsa-miR-181a-3p**	MIMAT0000270	0.0133	0.0516	-1.78
**hsa-miR-365b-3p**	MIMAT0022834	0.0146	0.0533	-2.08

**hsa-miR-125b-2-3p**	MIMAT0004603	0.0143	0.0533	19.14
hsa-miR-30a-3p	MIMAT0000088	0.0177	0.0626	1.45
hsa-miR-182-5p	MIMAT0000259	0.0184	0.0634	-1.59
hsa-miR-30e-5p	MIMAT0000692	0.0195	0.0653	1.97
hsa-let-7d-3p	MIMAT0004484	0.0209	0.0683	1.69
hsa-miR-195-5p	MIMAT0000461	0.0250	0.0796	2.00
hsa-miR-28-5p	MIMAT0000085	0.0259	0.0802	15.43
hsa-miR-29a-3p	MIMAT0000086	0.0289	0.0874	1.46
hsa-miR-335-5p	MIMAT0000765	0.0330	0.0974	10.14
hsa-miR-107	MIMAT0000104	0.0346	0.0997	1.49
hsa-miR-181a-2-3p	MIMAT0004558	0.0359	0.1010	-1.56
hsa-miR-16-5p	MIMAT0000069	0.0448	0.1219	1.65
hsa-miR-140-3p	MIMAT0004597	0.0452	0.1219	7.99
hsa-miR-532-5p	MIMAT0002888	0.0500	0.1342	1.61

Supplementary Table S2. Differentially regulated microRNA expression in diabetic limbus vs. central cornea (FDR adjusted p-value < 0.05)

ID	miRBase accession number	p-value	FDR-adjusted p-value	Fold Change
**hsa-miR-126-5p**	MIMAT0000444	6.48E-09	9.65E-07	55.02
**hsa-miR-143-3p**	MIMAT0000435	1.86E-08	1.39E-06	57.39
**hsa-miR-10a-5p**	MIMAT0000253	8.01E-08	3.98E-06	15.30
**hsa-miR-10b-5p**	MIMAT0000254	1.02E-06	3.78E-05	88.05
**hsa-miR-21-5p**	MIMAT0000076	1.27E-06	3.79E-05	3.61
**hsa-miR-25-3p**	MIMAT0000081	9.73E-05	0.0021	2.12
**hsa-miR-26b-5p**	MIMAT0000083	0.0001	0.0021	2.51
**hsa-miR-127-3p**	MIMAT0000446	0.0001	0.0024	74.44
**hsa-miR-126-3p**	MIMAT0000445	0.0001	0.0024	55.28
**hsa-let-7i-5p**	MIMAT0000415	0.0002	0.0024	2.53
**hsa-miR-184**	MIMAT0000454	0.0004	0.0053	-1.92
**hsa-miR-23b-3p**	MIMAT0000418	0.0005	0.0061	2.22
**hsa-miR-30a-5p**	MIMAT0000087	0.0006	0.0074	2.27
**hsa-miR-30a-3p**	MIMAT0000088	0.0011	0.0100	2.41
**hsa-miR-30d-5p**	MIMAT0000245	0.0011	0.0100	1.76
**hsa-miR-99a-5p**	MIMAT0000097	0.0011	0.0100	2.29
**hsa-miR-146a-5p**	MIMAT0000449	0.0015	0.0127	44.11
**hsa-miR-181c-5p**	MIMAT0000258	0.0017	0.0130	3.15
**hsa-miR-146b-5p**	MIMAT0002809	0.0020	0.0152	2.59
**hsa-miR-27b-3p**	MIMAT0000419	0.0022	0.0153	2.06
**hsa-miR-148b-3p**	MIMAT0000759	0.0026	0.0176	1.75
**hsa-miR-151a-3p**	MIMAT0000757	0.0028	0.0178	2.00
**hsa-miR-186-5p**	MIMAT0000456	0.0037	0.0203	1.87
**hsa-miR-26a-5p**	MIMAT0000082	0.0037	0.0203	2.03
**hsa-miR-148a-3p**	MIMAT0000243	0.0042	0.0203	1.43
**hsa-miR-501-3p**	MIMAT0004774	0.0054	0.0251	2.53
**hsa-let-7c-5p**	MIMAT0000064	0.0066	0.0287	2.01
**hsa-miR-199a-3p**	MIMAT0000232	0.0067	0.0287	2.36
**hsa-miR-199b-3p**	MIMAT0004563	0.0081	0.0329	2.41
**hsa-miR-192-5p**	MIMAT0000222	0.0084	0.0329	1.55
**hsa-miR-30e-5p**	MIMAT0000692	0.0086	0.0329	2.42
**hsa-miR-211-5p**	MIMAT0000268	0.0086	0.0329	27.61
**hsa-let-7b-5p**	MIMAT0000063	0.0099	0.0367	1.67
**hsa-miR-342-3p**	MIMAT0000753	0.0104	0.0367	1.85

**hsa-miR-19b-3p**	MIMAT0000074	0.0132	0.0456	22.04
**hsa-miR-107**	MIMAT0000104	0.0137	0.0462	2.05
hsa-miR-28-3p	MIMAT0004502	0.0188	0.0610	8.22
hsa-miR-130a-3p	MIMAT0000425	0.0241	0.0764	8.64
hsa-miR-24-3p	MIMAT0000080	0.0246	0.0764	10.03
hsa-miR-30b-5p	MIMAT0000420	0.0255	0.0775	8.10
hsa-miR-532-5p	MIMAT0002888	0.0317	0.0945	5.94
hsa-miR-374a-5p	MIMAT0000727	0.0342	0.0991	5.64
hsa-miR-92b-3p	MIMAT0003218	0.0350	0.0991	8.10
hsa-miR-21-3p	MIMAT0004494	0.0356	0.0991	6.91
hsa-miR-769-5p	MIMAT0003886	0.0359	0.0991	7.35
hsa-miR-221-3p	MIMAT0000278	0.0394	0.1068	7.98
hsa-miR-222-3p	MIMAT0000279	0.0407	0.1083	4.47
hsa-miR-30e-3p	MIMAT0000693	0.0469	0.1225	7.30
hsa-miR-92a-3p	MIMAT0000092	0.0478	0.1228	7.61
hsa-miR-29a-3p	MIMAT0000086	0.0500	0.1345	8.53

Supplementary Table S3. Differentially regulated microRNA expression in diabetic vs. normal limbus (p-value <0.05)

ID	miRBase accession number	p-value	Fold Change
**hsa-miR-365a-3p**	MIMAT0000710	0.002	-22.95
**hsa-miR-30a-3p**	MIMAT0000088	0.003	1.55
**hsa-miR-92b-3p**	MIMAT0003218	0.005	-1.52
**hsa-miR-10b-5p**	MIMAT0000254	0.010	1.59
**hsa-miR-3065-5p**	MIMAT0015066	0.013	2.71
**hsa-miR-214-3p**	MIMAT0000271	0.014	5.59
**hsa-miR-25-3p**	MIMAT0000081	0.017	1.39
**hsa-miR-425-5p**	MIMAT0003393	0.023	-4.11
**hsa-miR-328-3p**	MIMAT0000752	0.032	3.32
**hsa-miR-769-5p**	MIMAT0003886	0.035	1.40
**hsa-let-7d-3p**	MIMAT0004484	0.036	-2.57
**hsa-miR-155-5p**	MIMAT0000646	0.037	2.83
**hsa-miR-146a-5p**	MIMAT0000449	0.038	1.41
**hsa-miR-381-3p**	MIMAT0000736	0.038	3.01
**hsa-miR-3184-5p**	MIMAT0015064	0.040	-16.93
**hsa-miR-148b-3p**	MIMAT0000759	0.042	1.39
**hsa-miR-93-5p**	MIMAT0000093	0.044	-1.33
**hsa-miR-338-3p**	MIMAT0000763	0.046	-9.92
**hsa-miR-107**	MIMAT0000104	0.051	1.32
**hsa-miR-409-3p**	MIMAT0001639	0.053	3.30

Supplementary Table S4. Differentially regulated microRNA expression in T1DM vs. T2DM corneal limbus (FDR adjusted p-value < 0.05)

ID	microRNA accession number	p-value	FDR adjusted p-value	Fold Change T1D_Limbus vs T2D_Limbus)
**hsa-miR-125a-5p**	MIMAT0000443	8.80E-06	0.00043712	-3.31
**hsa-miR-99b-5p**	MIMAT0000689	1.64E-05	0.000489444	-2.48
**hsa-miR-10a-5p**	MIMAT0000253	0.000151002	0.00281241	6.07
**hsa-miR-100-5p**	MIMAT0000098	0.000349578	0.00520871	-2.86
**hsa-miR-191-5p**	MIMAT0000440	0.000347108	0.00520871	-1.42
**hsa-miR-25-3p**	MIMAT0000081	0.000411825	0.00557836	1.83
**hsa-miR-151a-3p**	MIMAT0000757	0.000461118	0.00570264	2.29
**hsa-miR-199b-5p**	MIMAT0000263	0.000607136	0.00646167	-2.99
**hsa-let-7i-5p**	MIMAT0000415	0.00107922	0.0107203	2.01
**hsa-miR-23a-3p**	MIMAT0000078	0.00183263	0.0160625	-1.62
**hsa-miR-28-3p**	MIMAT0004502	0.00178262	0.0160625	1.59
**hsa-miR-22-3p**	MIMAT0000077	0.00235824	0.019521	-1.74
**hsa-miR-24-3p**	MIMAT0000080	0.00266376	0.0208895	-2.30
**hsa-miR-30a-3p**	MIMAT0000088	0.00300506	0.0223877	1.97
**hsa-miR-10b-5p**	MIMAT0000254	0.00344579	0.0242926	2.27
**hsa-miR-342-3p**	MIMAT0000753	0.00374986	0.0242926	-1.42
**hsa-miR-30d-5p**	MIMAT0000245	0.0050217	0.0311764	-1.56
**hsa-miR-99a-5p**	MIMAT0000097	0.00869561	0.0479869	-1.53
**hsa-miR-186-5p**	MIMAT0000456	0.00916246	0.0487574	-1.82
hsa-miR-29c-3p	MIMAT0000681	0.0128246	0.0658917	-2.30
hsa-miR-125b-5p	MIMAT0000423	0.0152542	0.0703421	-1.81
hsa-miR-141-3p	MIMAT0000432	0.0153462	0.0703421	-2.39
hsa-miR-29a-3p	MIMAT0000086	0.0165233	0.0703421	-1.98

hsa-miR-423-3p	MIMAT0001340	0.0157835	0.0703421	-2.09
hsa-let-7d-5p	MIMAT0000065	0.0182103	0.0733995	-1.55
hsa-let-7g-5p	MIMAT0000414	0.0201917	0.0791726	-1.83
hsa-miR-106b-3p	MIMAT0004672	0.0224152	0.0844421	2.22
hsa-miR-200b-3p	MIMAT0000318	0.0242639	0.0848491	-1.90
hsa-miR-210-3p	MIMAT0000267	0.023436	0.0848491	-2.61
hsa-miR-23b-3p	MIMAT0000418	0.0244866	0.0848491	-1.53
hsa-miR-126-5p	MIMAT0000444	0.0271086	0.0917996	-1.63
hsa-miR-199a-3p	MIMAT0000232	0.033552	0.111094	-1.78
hsa-miR-27a-3p	MIMAT0000084	0.0358393	0.116088	-1.43
hsa-miR-192-5p	MIMAT0000222	0.0427578	0.132727	1.53
hsa-miR-126-3p	MIMAT0000445	0.0459036	0.133498	-1.67
hsa-miR-30e-3p	MIMAT0000693	0.0443316	0.133498	1.76
hsa-miR-21-3p	MIMAT0004494	0.0512584	0.13744	1.66
hsa-miR-769-5p	MIMAT0003886	0.0516551	0.13744	1.73
hsa-miR-98-5p	MIMAT0000096	0.0499349	0.13744	-1.37

Supplementary Table S5. Significant GO Terms of Predicted Targets of miR-10b-5p from miRTarBase

ID	Gene Ontology Term<br>Biological Process	P value	Only Validated miR-10 Targets and/or Epithelial-Related Genes Included
GO:0000122	Negative regulation of transcription from RNA polymerase II promoter	0.00085	PPARA, TP53, PAX6, HOXB3, STAT6, NOTCH1, TFAP2C, NCOR2, KLF4
GO:0048715	Negative regulation of oligodendrocyte differentiation	0.00107	NOTCH1, NF1
GO:0000082	G1/S transition of mitotic cell cycle	0.00135	CDKN1A, CDKN2A, CDK2
GO:0045944	Positive regulation of transcription from RNA polymerase II promoter	0.00144	PPARA, PAX6, HOXD10, STAT6, CDKN2A, TP53, DDX58, NOTCH1, TFAP2C, KLF4
GO:0045893	Positive regulation of transcription, DNA-templated	0.00194	PPARA, TP53, PAX6, FZD2, CDK2, NOTCH1, CDKN2A, KLF4
GO:0006351	Transcription, DNA-templated	0.00206	PAX6, HOXD1, HOXD10, HOXD11, STAT6, CSNK2A1, CDKN2A, POU2F2, TP53, HOXB3, TCF19, TCF15, NCOR2,
GO:0060070	Canonical Wnt signaling pathway	0.00242	SDC1, FZD2, PTEN, KLF4
GO:0060740	Prostate gland epithelium morphogenesis	0.00521	NOTCH1, RARG, ID4
GO:0007265	Ras protein signal transduction	0.00552	CDKN1A, CDKN2A, NF1, TP53, CDK2
GO:0006355	Regulation of transcription, DNA-templated	0.00739	PAX6, HOXD1, HOXD11, CSNK2A1, POU2F2, TP53, HOXB3, NOTCH1, ALKBH4
GO:0006915	Apoptotic process	0.01143	HTATIP2, TP53, PDCD4, PTEN, BCL2L11, CSNK2A1, CDKN2A, TIAM1, PPP1R13B,
GO:0006367	Transcription initiation from RNA polymerase II promoter	0.01202	PPARA, NOTCH1, RARG, NCOA6, NR4A3,
GO:0008284	Positive regulation of cell proliferation	0.01202	RARG, PTEN, CDK2, NOTCH1, CSNK2A1, TIAM1, ID4,
GO:0031065	Positive regulation of histone deacetylation	0.01306	SREBF1, CTBP1, TP53
GO:0031571	Mitotic G1 DNA damage checkpoint	0.013063	TP53, FBXO31, CDK2
GO:0003700	transcription factor activity, sequence-specific DNA binding	0.01380	PPARA, PAX6, HOXD10, STAT6, POU2F2, RARG, TP53, HOXB3, NOTCH1, TCF19, TFAP2C, TCF15, KLF4
GO:0090399	Replicative senescence	0.01551	CDKN1A, CDKN2A, TP53
GO:0008285	Negative regulation of cell proliferation	0.01633	CDC6, CTBP1, RARG, TP53, PTEN,

			NOTCH1, CDKN1A, CDKN2A, KLF4
GO:0045787	Positive regulation of cell cycle	0.01738	RPS15A, NR4A3, BCL2L11, C6ORF89
GO:0008134	Transcription factor binding	0.01799	PPARA, CTBP1, CDKN2A, GATA3, TP53, PAX6, FHL2, PPP1R13B, RORA, NCOR2
GO:0031100	Organ regeneration	0.02218	CDKN1A, NOTCH1, AXL, NR4A3, CDK2
GO:0043087	Regulation of GTPase activity	0.02218	EPHA4, RALBP1, NF1, PAFAH1B1, CRK
GO:0000079	Regulation of cyclin-dependent protein serine/threonine kinase activity	0.02338	CDC6, CDKN1A, CCNG1, PTEN
GO:0031647	Regulation of protein stability	0.02939	SREBF1, CDKN2A, USP3, LSS, PTEN
GO:0001649	Osteoblast differentiation	0.03335	SFRP1, NF1, FASN, FHL2, H3F3B, TPM4
GO:0001077	Transcriptional activator activity, RNA polymerase II core promoter proximal region sequence-specific binding	0.03695	SREBF1, PPARA, GATA3, ONECUT3, POU2F2, PAX6, TFAP2C, NR4A3, KLF4
GO:0001085	RNA polymerase II transcription factor binding	0.04070	GATA3, TP53, ID4, KLF4
GO:1902263	Apoptotic process involved in embryonic digit morphogenesis	0.04783	NOTCH1, BCL2L11

Supplementary Table S6. Primers used for QRT-PCR

Gene	Sequence (forward & reverse, 5'-3')	Accession number	Amplicon size
β2-Microglobulin<br>Integrated DNA Tech.	GGA CTG GTC TTT CTA TCT CTT GT	NM_004048.2	143
B2M<br>HS.PT.58V.18759587	ACC TCC ATG ATG CTG CTT AC		
β-Actin<br>Integrated DNA Tech.	GTC CCC CAA CTT GAG ATG TAT G	NM_001101.3	83
β-Actin<br>HS.PT.39A.22214847	AAG TCA GTG TAC AGG TAA GCC		
SOX9	GAC TTC CGC GAC GTG GAC	NM_000346.3	99
SOX9	GTT GGG CGG CAG GTA CTG		
OCT-4	GGG AAG GTA TTC AGC CAA AC	NM_001173531.2	145
OCT-4	CTG CTT TGC ATA TCT CCT GAA G		
N-Cadherin	CAA GAG CTT GTC AGG ATC AG	NM_001792.4	184
CDH2	CAT GTG CCC TCA AAT GAA AC		
PAX6	CCT GGA GAA AGA GTT TGA GAG	NM_001258464.1	124
PAX6	CCA TTT GGC CCT TCG ATT A		
Keratin 3	GAA GAA GGA TGT GGA CAG TG	NM_057088.2	178
KRT3	GTC CAG GGA GCG ATT ATT G		
Keratin 12	GCT GAA GCC TGG TTC ATT	NM_000223.3	156
KRT12	TTT CTT CAT GGC GAG CTG		
Keratin 14	CCT GCT GAG ATC AAA GAC TAC	NM_000526.4	150
KRT14	CTC ATA CTT GGT GCG GAA G		
Keratin 15	CGA AGA GGA GAT GAA GGA GTT C	NM_002275.3	226
KRT15	CTT GCT GGT CTG GAT CAT TTC		
Keratin 18	TCA GAG ACT GGA GCC ATT A	NM_000224.2	149
KRT18	CAG CTC TGT CTC ATA CTT GAC		
Keratin 17	TGA GCT GGA GGT GAA GAT	NM_000422.2	200

KRT17	GCT CTG TCT CAA ACT TGG T		
Vimentin	CAG GAT TTC TCT GCC TCT TC	NM_003380.3	143
VIM	CGT TGA TAA CCT GTC CAT CTC		
DKK1	TGG AAT ATG TGT GTC TTC TGA T	NM_012242.2	157
DKK1	AGA ACC TTC TTG TCC TTT GG		
EPHB2	TCC TCT GGC ATC AAC CT	NM_017449.4	165
EPHB2	CTC CAT CAT CAT CTG AGA CAC		
P4HB	GAA TAT ACA GCT GGC AGA GAG	NM_000918.3	154
P4HB	ACT CCA CGT CCT TGA AGA		
HBEGF	GTG CTG TCA TCT GTC TGT C	NM_001945.2	151
HBEGF	CAG CCG ATT CCT TGA GC		
EDEM1	CTT TGA GGC CAC GAT AAG G	NM_014674.2	199
EDEM1	TAG ATT CAC CCG AGG ATA TGG		
KLF4	CAC ATT AAT GAG GCA GCC A	NM_004235.5	166
KLF4	TCG CTT CAT GTG GGA GA		
PTEN	CGG AAC TTG CAA TCC TCA G	NM_000314.6	136
PTEN	GAT ATC ACC ACA CAC AGG TAA C		
GSK3B	CTC CAG TGG TGA GAA GAA AG	NM_001146156.1	153
GSK3B	CTT CGG AAC AGC TGA TAC AT		
TP53	GCA CTA AGC GAG CAC TG	NM_001276695.1	119
TP53	CAG GAA GTA ACA CCA TCG TAA G		
ΔNp63	TGT ACC TGG AAA ACA ATG CCC A	NM_001114982.1	103
ΔNp63	GAC GAG GAG CCG TTC TGA ATC T		
WNT7A	GCCAAGGTCTTTGTGGATGC	NM_004625.3	99
WNT7A	GTTCTCCTCCAGGATCTTTCGG		
